# Chemokine expression predicts T cell-inflammation and improved survival with checkpoint inhibition across solid cancers

**DOI:** 10.1038/s41698-023-00428-2

**Published:** 2023-08-09

**Authors:** Joan Miguel Romero, Emma Titmuss, Yifan Wang, James Vafiadis, Alain Pacis, Gun Ho Jang, Amy Zhang, Bryn Golesworthy, Tatiana Lenko, Laura M. Williamson, Barbara Grünwald, Grainne M. O’Kane, Steven J. M. Jones, Marco. A. Marra, Julie M. Wilson, Steven Gallinger, Janessa Laskin, George Zogopoulos

**Affiliations:** 1 Research Institute of the McGill University Health Centre, Montréal, QC Canada; 2 Rosalind and Morris Goodman Cancer Institute of McGill University, Montréal, QC Canada; 3 Canada’s Michael Smith Genome Sciences Centre at BC Cancer, Vancouver, BC Canada; 4 Department of Surgery, McGill University, Montréal, QC Canada; 5 Canadian Centre for Computational Genomics, McGill University and Genome Québec Innovation Centre, Montréal, QC Canada; 6 PanCuRx Translational Research Initiative, Ontario Institute for Cancer Research, Toronto, ON Canada; 7 Princess Margaret Cancer Centre, University Health Network, University of Toronto, Toronto, ON Canada; 8 Wallace McCain Centre for Pancreatic Cancer, Princess Margaret Cancer Centre, Toronto, ON Canada; 9 Department of Medical Genetics, University of British Columbia, Vancouver, BC Canada; 10 Department of Medical Oncology, BC Cancer, Vancouver, BC Canada; 11 Department of Oncology, McGill University, Montréal, QC Canada

**Keywords:** Prognostic markers, Tumour immunology, Cancer genomics, Cancer microenvironment

## Abstract

Immune checkpoint inhibitors (ICI) are highly effective in specific cancers where canonical markers of antitumor immunity are used for patient selection. Improved predictors of T cell-inflammation are needed to identify ICI-responsive tumor subsets in additional cancer types. We investigated associations of a 4-chemokine expression signature (c-Score: *CCL4*, *CCL5*, *CXCL9*, *CXCL10*) with metrics of antitumor immunity across tumor types. Across cancer entities from The Cancer Genome Atlas, subgroups of tumors displayed high expression of the c-Score (c-Score^hi^) with increased expression of immune checkpoint (IC) genes and transcriptional hallmarks of the cancer-immunity cycle. There was an incomplete association of the c-Score with high tumor mutation burden (TMB), with only 15% of c-Score^hi^ tumors displaying ≥10 mutations per megabase. In a heterogeneous pan-cancer cohort of 82 patients, with advanced and previously treated solid cancers, c-Score^hi^ tumors had a longer median time to progression (103 *versus* 72 days, *P* = 0.012) and overall survival (382 *versus* 196 days, *P* = 0.038) following ICI therapy initiation, compared to patients with low c-Score expression. We also found c-Score stratification to outperform TMB assignment for overall survival prediction (HR = 0.42 [0.22–0.79], *P* = 0.008 *versus* HR = 0.60 [0.29-1.27], *P* = 0.18, respectively). Assessment of the c-Score using the TIDE and PredictIO databases, which include ICI treatment outcomes from 10 tumor types, provided further support for the c-Score as a predictive ICI therapeutic biomarker. In summary, the c-Score identifies patients with hallmarks of T cell-inflammation and potential response to ICI treatment across cancer types, which is missed by TMB assignment.

## Introduction

Antitumoral CD8^+^ T cells mediate immune-checkpoint inhibitor (ICI) response^1–3^. Chemokines have central roles in antitumoral immune cell infiltration, including recruitment of dendritic cells and antigen-specific CD8^+^ T cells^4,5^. We have identified a 4-chemokine transcriptomic signature (c-Score: *CCL4*, *CCL5*, *CXCL9*, *CXCL10*) to associate with T cell-inflammation in pancreatic cancer^6^. All four chemokines have roles in recruiting immune cells critical to cancer immunity. *CCL4* mitigates dendritic cell migration and subsequent T cell activation and tumor infiltration^7^, while *CCL5, CXCL9*, and *CXCL10* are associated with T cell infiltration across solid tumors^8–15^.

High mutational burden (TMB) in mismatch repair (MMR) deficient (MMRD) tumors enhances immune antitumor responses^16–19^. Similarly, tumors with homologous recombination (HR) repair pathway deficiencies (HRD) exhibit increased mutations and may also harbor a genetically favorable milieu to elicit an antitumor immune response in HRD-associated cancers^20,21^. In metastatic pancreaticobiliary cancers with HRD, responders to ICI therapy express higher levels of *CCL4*, *CXCL9*, and *CXCL10*^22^.

Elevated TMB (≥10 mutations/megabase) has emerged as a tumor-agnostic FDA-approved biomarker for ICI therapy^23^. The transformative treatment benefits of ICIs in a select subset of malignancies has triggered new research to identify improved biomarkers that will identify T cell-inflamed cancer subpopulations within TMB low and broadly immune cold cancer types^24^. In this study, we investigated the predictiveness of the 4-chemokine c-Score in revealing T cell-inflammation across 25 tumor types by leveraging genomic and transcriptomic data from 6455 patients. Using a real-world clinical cohort of 82 advanced cancer patients with ICI response data, we subsequently evaluated the ability of the 4-chemokine signature to predict ICI treatment response compared to TMB.

## Results

**Fig. 1:**
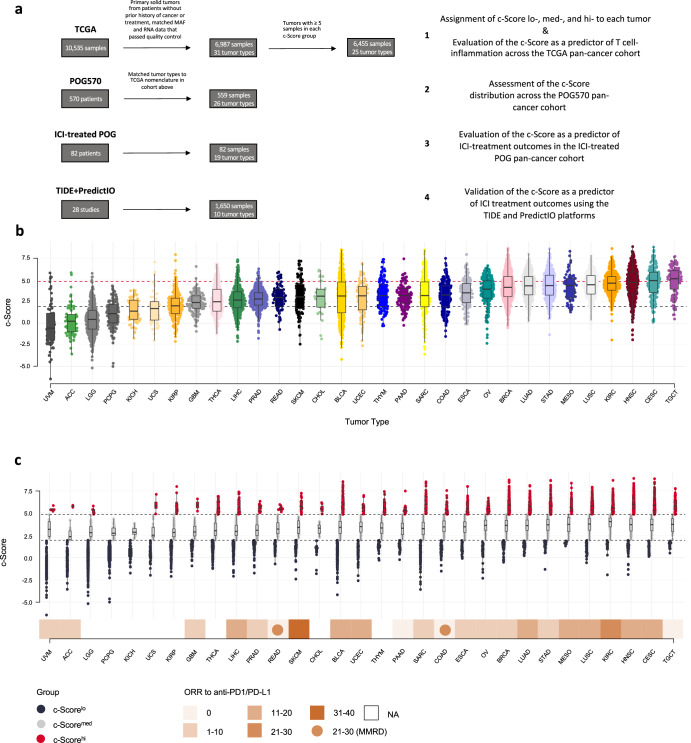
Expression of the 4-chemokine signature across tumor types. **a** Consort diagram describing principal cohorts and analysis pipeline. **b** 4-chemokine signature (c-Score), calculated as a mean expression of *CCL4*, *CCL5*, *CXCL9*, and *CXCL10*, across 31 tumor types from TCGA. Lowest to highest median c-Score plotted from left to right. Red and blue dashed lines represent top and bottom quartile cut offs, respectively. **c** Distribution of c-Score, with segregation according to c-Score^hi^ (*n* = 1747), c-Score^med^ (*n* = 3493) and c-Score^lo^ (*n* = 1747) expression profiles. Expression profile scoring is based on the bottom, middle and top quartiles across the entire dataset. The bottom track displays the reported objective response rate to anti-PD1 or anti-PD-L1 immune checkpoint therapy^27^. Circles = ORR in MMRD tumors for that tumor subtype. NA = ORR not available. Median, quartiles, minimum and maximum values are represented by the central line, limits of box, and ends of lines of boxplots shown in (**b** and **c**).

Two discovery pan-cancer datasets were used to examine the 4-chemokine signature (*CCL4, CCL5, CXCL9, CXCL10*; herein referred to as c-Score) as a predictive biomarker for T cell-inflammation and ICI treatment outcomes across different solid tumor types (Fig. 1). The utility of the c-Score in predicting ICI treatment outcomes was then validated using two additional databases (TIDE^25^, PredictIO^26^), which include 28 studies with RNA sequencing and ICI response data from 10 tumor types (Fig. 1).

### A wide range of tumor types across TCGA cohort have high expression of the 4-chemokine signature

We analyzed transcriptomic cancer data from The Cancer Genome Atlas (TCGA). The cohort consisted of 31 tumor types from 6987 patients (histological subtypes and abbreviations found in Supplementary Data File 1). Uveal melanoma (UVM), adrenocortical carcinoma (ACC), and lower grade glioma (LGG) had the lowest c-Score expression, while testicular germ cell (TGCT), cervical squamous cell carcinoma (CESC) and head and neck squamous cell carcinoma (HNSC) were the highest expressors of the c-Score (Fig. 1b). To compare differences in T cell-inflammation within and between tumor types, c-Score expression was categorized for each tumor type in quartile distributions based on expression of the 4 chemokines across all 6987 patients in TCGA cohort. Tumors having c-Score expression equal or greater than the third quartile were classified as c-Score^hi^ (*n* = 1747), those expressing equal or less than the first quartile were classified as c-Score^lo^ (*n* = 1747), and those with scores in between were classified as c-Score^med^ (*n* = 3493). Across these 31 tumor types, only two (pheochromocytoma [PCPG], and kidney chromophobe [KICH]) were devoid of c-Score^hi^ tumors. An additional four tumor types (UVM, ACC, uterine carcinosarcoma [UCS], glioblastoma multiforme [GBM]) with a paucity of c-Score^hi^ tumors were excluded, leaving 25 tumor types from 6455 patients for downstream analyses comparing c-Score groups (Methods, Fig. 1). Tumor types with reported objective response rates (ORR) of >11 to anti-PD-1 or PD-L1 therapies^27^ included top c-Score expressors (mesothelioma [MESO], lung squamous cell carcinoma [LUSC], kidney renal clear cell carcinoma [KIRC], HNSC, CESC), though other modest and strong responders to ICIs were seen throughout the c-Score expression spectrum (Fig. 1c). Interestingly, TGCT, the highest c-Score expressor, had low ORR to ICIs^27^. For data representation across metrics of T cell-inflammation and antitumor immunity, the c-Score^lo^ and c-Score^med^ cases were grouped together (c-Score^lo+med^) and compared to the c-Score^hi^ group.

### Tumors in TCGA cohort with high expression of the c-Score display hallmarks of T cell-inflammation

**Fig. 2:**
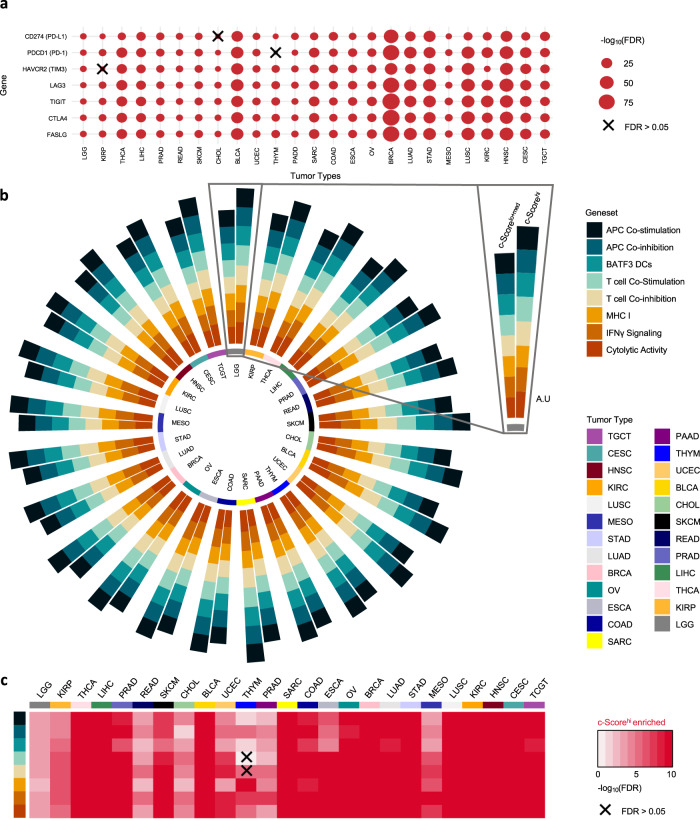
c-Score^hi^ tumors are enriched in genes and pathways involved with the cancer-immunity cycle. **a** Degree of the significance of immunoregulatory gene expression between c-Score^hi^ *versus* c-Score^lo+med^ groups across 25 tumor types from TCGA. Wilcoxon rank-sum test, FDR adjusted. (**b**) Scores for and (**c**) degree of significance in eight genesets representing processes involved in the cancer-immunity cycle across c-Score^hi^ (*n* = 1735) and c-Score^lo+med^ (*n* = 4720) tumors in this dataset. For each pair, c-Score^lo+med^ tumors are on left, c-Score^hi^ tumors are on the right, as depicted in the inset. A.U. Arbitrary units, translated onto a positive scale (see Methods). Wilcoxon rank-sum test, FDR adjusted. X represents FDR > 0.05.

To investigate the immune profiles of c-Score^hi^ tumors across the 25 TCGA tumor types, we evaluated immunomodulatory genes involved in immune checkpoint blockade response, (*CD274* [*PD-L1*], *PDCD1* [*PD-1*], *HAVCR2* [*TIM3*], *LAG3*, *TIGIT*, *CTLA4*, and *FASLG*). Across tumor types, these genes were overexpressed in c-Score^hi^ tumors *versus* c-Score^lo+med^ tumors, except for cholangiocarcinoma (CHOL), thymoma (THYM), and kidney papillary renal cell carcinoma (KIRP) that did not meet the false discovery rate (FDR) threshold for *CD274*, *PDCD1*, and *HAVCR2*, respectively (Fig. 2a; Wilcoxon rank-sum, FDR adjusted). *CD40*, which encodes a key molecule involved in antigen presentation^2,28–30^, was also upregulated in the c-Score^hi^ tumors across tumor types (Supplementary Fig. 1a; 92%, 23/25).

To expose higher-dimensional differences in the transcriptomic immune profiles of these tumors, we compared immune cell proportions and gene set differences between the two c-Score groups. Compared to c-Score^lo+med^, c-Score^hi^ tumors had increased transcriptional patterns associated with the presence of M1-type macrophages, CD8^+^ T cells, and T Regulatory cells in most tumor types (Supplementary Fig. 1b; enriched in 96%, 88%, 64% of tumor types, respectively, Wilcoxon rank-sum, FDR adjusted). We then examined eight genesets representative of processes key to the cancer-immunity cycle, including co-stimulation and inhibition of antigen presenting cells (APCs), tumor antigen cross-presenting BATF3 dendritic cell (DC) expression, co-stimulation and inhibition of T cells, major histocompatibility class I (MHC I) molecule expression, interferon gamma signaling, and cytolytic activity^7,31,32^ (Fig. 2b). Compared to c-Score^lo+med^, c-Score^hi^ tumors had increased expression of at least six genesets, with 96% (24/25) of tumor types having increased expression of all genesets (Fig. 2c; Wilcoxon rank-sum, FDR adjusted). Similarly, genesets predictive of T cell-inflammation^33^ and response to ICI^34^ were upregulated in c-Score^hi^ tumors across these 25 tumor types (Supplementary Fig. 1c; Wilcoxon rank-sum, FDR adjusted). Together, these results show a correlation between high c-Score expression and transcriptional hallmarks of T cell-inflammation across tumor types.

### High proportion of tumors in TCGA cohort are c-Score^hi^ despite low TMB

**Fig. 3:**
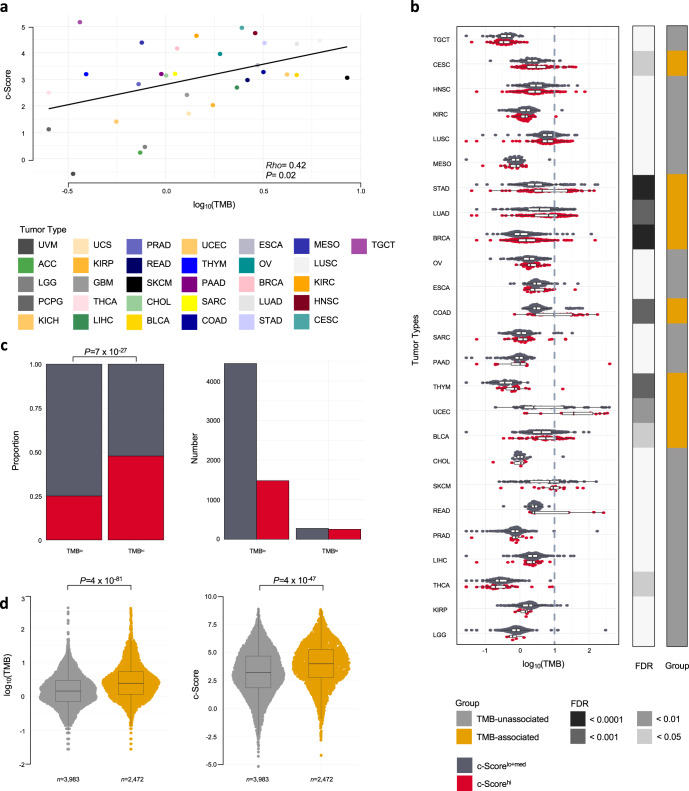
A high proportion of tumors are c-Score^hi^ despite low TMB. **a** Spearman correlation of median TMB *versus* c-Score across all 31 tumor types from TCGA. **b** Association between TMB and c-Score signature across 25 tumor types from TCGA. Boxplots depict a comparison of TMB in c-Score^hi^ *versus* c-Score^lo+med^ tumors, across tumor types. The left track displays FDR adjusted *P* values of TMB differences between groups. Right track displays group designation. The dashed line shows TMB threshold of 10 mutations/megabase. Wilcoxon rank-sum test, FDR adjusted. **c** The proportion (left) and absolute number (right) of c-Score^hi^ and c-Score^lo+med^ tumors in TMB^lo^ *versus* TMB^hi^ (TMB ≥ 10) cases across 25 tumor types. Fisher’s exact test. **d** Tumor types where c-Score^hi^ tumors had higher TMB versus c-Score^lo+med^ were classified as ‘TMB-associated’. TMB (left) and the c-Score (right) in ‘TMB-unassociated’ *versus* ‘TMB-associated’. Wilcoxon rank-sum test. Median, quartiles, minimum and maximum values are represented by the central line, limits of box, and ends of lines of boxplots shown in b and d.

Tumors displaying high TMB are presumed to be more immunogenic due to their increased levels of immune-inciting neoantigens^27,35,36^. However, the association of TMB and markers of T cell-inflammation appears to be specific to certain tumor types and is not observed in others^6,37–39^. Therefore, we evaluated whether the c-Score identifies T cell-inflammation that is missed by the TMB surrogate biomarker. We observed a relationship between median TMB and c-Score expression across tumor types (Fig. 3a; *Rho* = 0.42, *P* = 0.020, Spearman correlation, all 31 TCGA tumor types). However, of the 25 tumor types analyzed, only 32% (8/25; CESC, stomach adenocarcinoma [STAD], lung adenocarcinoma [LUAD], breast carcinoma [BRCA], colon adenocarcinoma [COAD], THYM, endometroid endometrial adenocarcinoma [UCEC], and bladder urothelial carcinoma [BLCA]) had higher TMB in the c-Score^hi^ group *versus* the c-Score^lo+med^ group (Fig. 3b; *P* < 0.05, Wilcoxon rank-sum, FDR adjusted). A similar relationship was also observed for single nucleotide variant (SNV)-derived neoantigens (Supplementary Fig. 2a). Notably, among the 25 histological tumor types, high TMB tumors (TMB^hi^, *n* = 530; ≥10 mutations/megabase) were c-Score^hi^ enriched compared to TMB^lo^ (*n* = 5925) tumors (Fig. 3c; 48% *versus* 25%, *P* < 0.001, Fisher’s exact test). However, TMB^hi^ tumors only represented 15% of the total c-Score^hi^ tumors across histological types (Fig. 3c), suggesting an incomplete association of TMB and the c-Score.

We, therefore, classified histological tumor types with higher TMB in the c-Score^hi^ group *versus* the c-Score^lo-med^ group as ‘TMB-associated’. Only eight of the 25 histological tumor types were ‘TMB-associated’, whereas the remaining 17 tumor types were ‘TMB-unassociated*’* (Fig. 3b). The eight ‘TMB-associated’ tumor types (CESC, STAD, LUAD, BRCA, COAD, THYM, UCEC, BLCA) displayed elevated TMB (*P* < 0.001) and c-Score (*P* < 0.001) expression (Fig. 3d; Wilcoxon rank-sum), with a higher proportion of tumors meeting the c-Score^hi^ designation (Supplementary Fig. 2b; *P* < 0.001, Fisher’s exact test). Importantly, 52% (*n* = 902/1735) of all c-Score^hi^ tumors across histological tumor types were classified as ‘TMB-unassociated’ (Supplementary Fig. 2b).

Next, we compared these tumors across histological types based on their c-Score group and classification as TMB^hi^ or TMB^lo^. Considering both c-Score and TMB assignments, we found that c-Score^hi^TMB^hi^ tumors had increased expression of most cancer-immunity cycle gene sets and marginally increased immune checkpoint (IC) gene expression compared to the c-Score^hi^TMB^lo^ group (Supplementary Fig. 2c, d; Wilcoxon rank-sum, FDR adjusted, all 31 TCGA tumor types). These observations suggest that tumors with both high expression of the c-Score and a TMB ≥ 10 mutations/megabase have the strongest antitumor immunity phenotype. However, c-Score^hi^TMB^hi^ tumors represent only 3.6% of tumors across the total cohort, with a larger fraction of c-Score^hi^ tumors having TMB^lo^ scores (Fig. 3c). Therefore, c-Score^hi^ identifies T cell-inflammation, with potential immune checkpoint treatment implications, that is not captured by TMB assignment.

### Tumors with high c-Score expression in the ICI-treated POG cohort respond to immune checkpoint blockade

To explore the prognostic value of the c-Score in immunotherapy response, we used previously published transcriptome sequencing data from the Personalized OncoGenomics (POG570) program^40^. We first evaluated the c-Score distribution across one cohort of patients from the POG program (POG570, *n* = 559 patients, 26 tumor types)^41^. Similar to the distribution in TCGA, c-Score^hi^ tumors were present in most tumor types (73%, *n* = 19/26, Supplementary Fig. 3a). Notably, in both POG570 and TCGA, the same tumor types had overall lower c-Score expression values (ACC, UVM, thyroid carcinoma [THCA]) *versus* higher values (ovarian serous cystadenocarcinoma [OV], CESC and MESO).

**Fig. 4:**
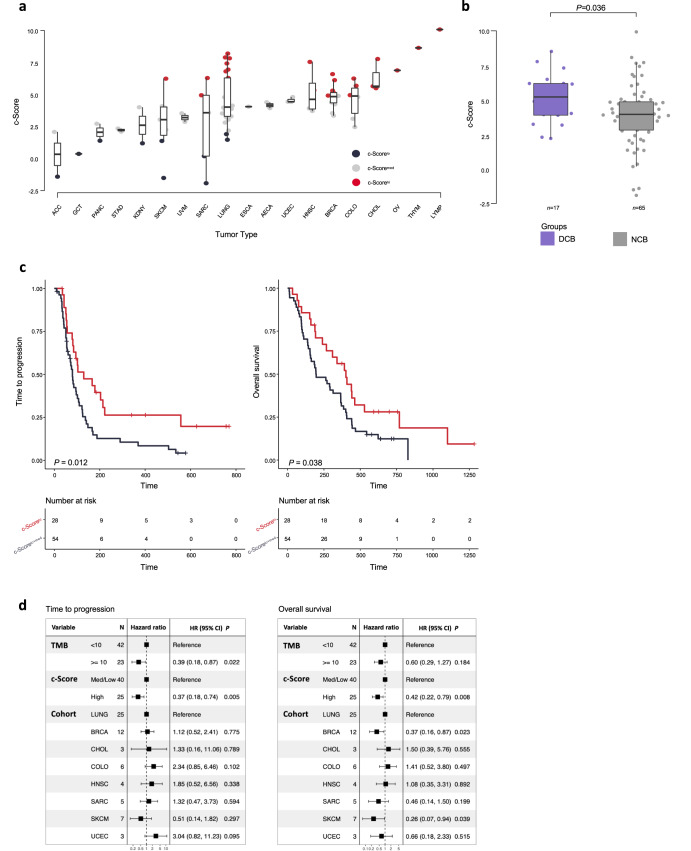
Tumors with high expression of the c-Score display increased response to immune checkpoint inhibition. **a** Distribution of c-Score^hi^, c-Score^med^, and c-Score^lo^ groups across ICI treated patients in the POG program. **b** c-Score expression in patients with durable clinical benefit (DCB, *n* = 17) *versus* no clinical benefit (NCB, *n* = 65). Wilcoxon rank-sum test. Median, quartiles, minimum and maximum values are represented by the central line, limits of box, and ends of lines of boxplots shown. **c** Time to progression and overall survival for patients stratified by c-Score group. *P* values indicate log-rank tests between the c-Score^hi^ and c-Score^lo+med^ groups. **d** Multivariate cox proportional hazards models for time to progression and overall survival. Tumor types with at least 3 patients were included in the models (total of 65 patients). Error bars represent 95% CI.

Next, we evaluated the relationship of the c-Score with ICI therapy outcomes in an ICI-treated POG cohort^40^. This heterogenous pan-cancer cohort of 82 patients, with advanced and previously treated solid cancers, received ICI therapy following tumor biopsy for whole transcriptomic sequencing. Using the transcriptomic data from their tumor biopsies, the 82 patients were stratified into c-Score^hi^ (*n* = 28), c-Score^med^ (*n* = 44), and c-Score^lo^ (*n* = 10) groups based on the thresholds determined using TCGA data (Fig. 4a; Methods). Patients who exhibited durable clinical benefit (DCB) on treatment had higher c-Score expression (Fig. 4b; Wilcoxon rank-sum), and this observation remained significant after correcting for differences in histological tumor type (*P* = 0.036, multivariate linear regression). Thirty-six percent (10/28) of patients in the c-Score^hi^ group had DCB, compared to 16% (7/44) for c-Score^med^ and 0% (0/10) for the c-Score^lo^ group (Supplementary Fig. 3b; *P* = 0.029, chi-squared). Furthermore, patients with high expression of the c-Score had a longer time to progression (TTP, median 103 *versus* 72 days, *P* = 0.012) and overall survival (OS, median 382 *versus* 196 days, *P* = 0.038, log-rank) compared to the c-Score^lo+med^ group (Fig. 4c, d). Comparison of the c-Score^hi^, c-Score^med^ and c-Score^lo^ groups also showed parallel TTP (*P* = 0.012) and OS (*P* = 0.017, log-rank) clinical outcome differences (Supplementary Fig. 3c). The c-Score^hi^ association with favorable outcomes was independent of tumor type (Fig. 4d; TTP hazard ratio (HR) = 0.37 [0.18-0.74], *P* = 0.005; OS HR = 0.42 [0.22-0.79], *P* = 0.008, Cox proportional hazards).

Since expression of IC genes has been associated with ICI treatment outcomes^32,34,35^, we examined the ICI treatment response predictiveness of *CD274, PDCD1*, and *CTLA4* in the ICI-treated POG cohort. For each IC gene, expression was stratified into high and low/medium quartiles, similar to c-Score group assignments. Evaluating each IC gene individually in a cox proportional hazards model, along with c-Score, TMB and tumor type, none of the three IC genes were predictive of TTP or OS. The c-Score, however, remained strongly predictive of TTP and OS (Supplementary Fig. 3d).

We then compared the predictiveness of c-Score *versus* TMB assignments on TTP and OS. The c-Score^hi^ biomarker performed equally to TMB^hi^ assignments in predicting TTP (HR = 0.37 [0.18–0.74], *P* = 0.005 *versus* HR = 0.39 [0.18–0.87], *P* = 0.022, respectively, Cox proportional hazards) and outperformed TMB when comparing OS (Fig. 4d; HR = 0.42 [0.22-0.79], *P* = 0.008 *versus* HR = 0.60 [0.29-1.27], *P* = 0.18, respectively, Cox proportional hazards). In a subgroup analysis, c-Score^hi^TMB^hi^ patients had a longer TTP compared to the other subgroups (*P* = 0.00092). However, an OS difference between the four groups (c-Score^hi^TMB^hi^, c-Score^lo^TMB^hi^, c-Score^hi^TMB^lo^, c-Score^lo^TMB^lo^) was not observed (Supplementary Fig. 3e*; P* = 0.094, log-rank). Moreover, no difference in OS between c-Score^hi^ *versus* c-Score^lo+med^ patients was observed across three independent ICI-untreated cohorts (data not shown: PanCuRx^20^, treatment naïve resectable pancreatic adenocarcinoma (PAAD) cohort, *n* = 109, *P* = 0.68; COMPASS^42^, treatment naïve PAAD metastatic cohort, *n* = 117, *P* = 0.48; POG570, entire pan-cancer non-immunotherapy treated cohort, *n* = 402, *P* = 0.3). Furthermore, when survival data included time points before patients received ICI (date of advanced disease *versus* date of ICI initiation), the prognostic ability of the c-Score score was lost (Supplementary Fig. 3f; *P* = 0.29, log-rank).

### Validation of the c-Score as a predictor of immune checkpoint blockade treatment response in additional cohorts

To further validate the c-Score, we first used the TIDE platform^25^ containing 25 databases from 19 studies spanning 7 tumor types (*n* = 1290). The c-Score had a positive predictive value of ICI response in 72% (18/25) of the datasets analyzed. Moreover, the c-Score predicted ICI response with an AUC ≥ 0.8 in 24% (6/25) of the datasets, compared to 4% (1/25) using the TIDE computational framework model (Supplementary Fig. 4a). Next, we used the PredictIO platform^26^, encompassing 15 databases from 15 studies spanning eight tumor types (*n* = 715). The c-Score predicted treatment response, OS, and progression free survival in a pooled analysis of all datasets (Supplementary Fig. 4b–d, left panels). Furthermore, the c-Score was significantly superior at predicting treatment response, OS, and progression free survival compared to 37 published ICI biomarker signatures, second only to the 100-gene PredictIO gene signature (Supplementary Fig. 4b–d, right panels). These two validation analyses further support the clinical utility of the c-Score for predicting ICI treatment outcomes.

### c-Score expression associates with both DNA repair deficient and proficient tumors

**Fig. 5:**
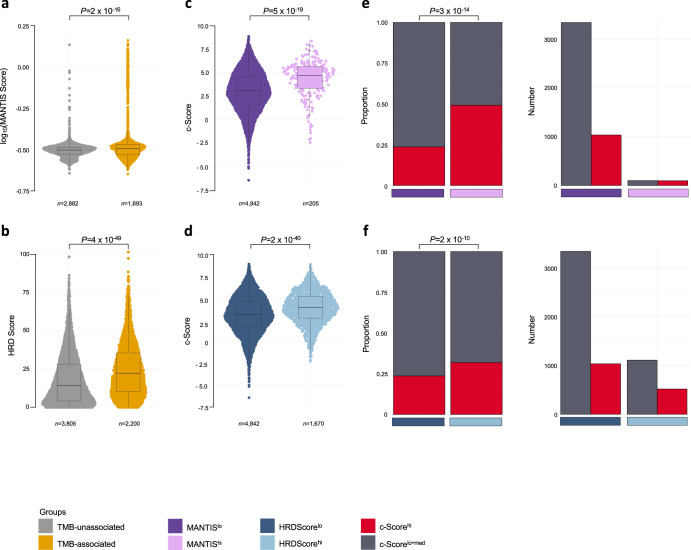
Tumors with defects in DNA damage repair display elevated expression of the c-Score. **a** MANTIS scores, excluding HRDScore^hi^ tumors, and (**b**) HRD scores, excluding MANTIS^hi^ tumors, in ‘TMB-unassociated *versus* ‘TMB-associated’ tumors across 25 tumor types from TCGA. Boxplots depicting the c-Score in (**c**), MANTIS^lo^ *versus* MANTIS^hi^ tumors and (**d**), HRDScore^lo^ *versus* HRDScore^hi^ tumors, excluding HRDScore^hi^ and MANTIS^hi^ tumors, respectively, from this cohort. Wilcoxon rank-sum test. The proportion (left) and absolute number (right) of c-Score^hi^ and c-Score^lo+med^ tumors in (**e**), MANTIS^lo^ (*n* = 4376) *versus* MANTIS^hi^ (*n* = 199) and (**f**), HRDScore^lo^ (*n* = 4376) *versus* HRDScore^hi^ (*n* = 1629) tumors from this cohort. Fisher’s exact test. Median, quartiles, minimum and maximum values are represented by the central line, limits of box, and ends of lines of boxplots shown in **a**–**d**.

We next examined whether the incomplete association between TMB and the c-Score could be explained by genomic differences. Patients with tumors defective in DNA repair and fidelity pathways, including MMRD, HRD, and the *POLE*/*POLD1* genes, have been shown to respond favorably to ICIs and display increased hallmarks of T cell-inflammation^20,21,43,44^. Mechanisms leading to high TMB may explain an association between TMB and antitumoral immune activity. For example, defects in DNA repair, such as in MMRD, stimulate innate immune signaling pathways^45,46^. Therefore, we investigated whether tumors that had an association of high TMB with the c-Score (*i.e*., ‘TMB-associated’) had mutations in genes involved with DNA repair pathways across the 25 TCGA tumor types. Specifically, we considered mutations in DNA mismatch repair (*MLH1*, *MSH2*, *MSH6*, *PMS2*), homologous recombination repair (*BRCA1*, *BRCA2*, *PALB2*), and DNA replication and proofreading (*POLD1*, *POLE*) genes, as well as molecular hallmarks indicative of mutations in these pathways (*i.e*., for MMRD, microsatellite instability (MSI) inferred using MANTIS scores^47^; for HRD, HRDScore^48,49^). Indeed, ‘TMB-associated’ tumor types had a higher frequency of mutations, including protein-truncating mutations, in these DNA integrity pathway genes (Supplementary Fig. 5a–f; Fisher’s exact test) and increased MSI and HRD scores (Fig. 5a, b; Wilcoxon rank-sum). We then stratified tumors into MSI high and low (MANTIS^hi^, *n* = 205; MANTIS^lo^, *n* = 4842) and HRD high and low (HRDScore^hi^, *n* = 1670; HRDScore^lo^, *n* = 4842; Methods, Supplementary Fig. 5g–i). MSI^hi^ tumors displayed increased expression of the c-Score (Fig. 5c; *P* = 5 × 10^−19^, Wilcoxon rank-sum), in agreement with previous reports that MSI tumors harbor immunogenicity^43^. While HRD^hi^ tumors also had increased expression of the c-Score, the association was lost after controlling for tumor type (Fig. 5d; Wilcoxon rank-sum, controlled using multivariate linear regression). Thus, HRD tumors may display increased T cell-inflammation that is both weaker compared to MMRD tumors and more variable among histological tumor types.

Among the full 31 TCGA tumor type dataset, the MSI score was positively associated with the c-Score only in COAD (Supplementary Fig. 6a; *Rho* = 0.35, *P* < 0.001, Spearman correlation, FDR adjusted). The HRDScore was positively correlated with the c-Score in THYM (*Rho* = 0.33, *P* < 0.01) and negatively correlated in HNSC (Supplementary Fig. 6b; *Rho* = −0.36, *P* < 0.01, Spearman correlation, FDR adjusted). These results suggest continuous metrics of MSI or HRD correlate linearly with the c-Score only in a subset of tumor types, and analyses using dichotomous classifications of these pathway defects may highlight further correlations with the c-Score across tumors.

Similar to their correlation with TMB, TCGA tumors harboring DNA repair pathway defects were enriched for c-Score^hi^ expression (Fig. 5e, f; 49% *versus* 24%, *P* = 3 × 10^−14^ for MSI; 32% *versus* 24%, *P* = 2 × 10^−10^ for HRD, Fisher’s exact test). However, the majority of c-Score^hi^ tumors had low MSI and HRD scores (Fig. 5e, f; 91% and 67%, respectively). HRDetect^hi^ (*n* = 8) and MSIsensor^hi^ (*n* = 3) tumors in POG570 cohort had higher median c-Score expression than their low counterparts, which was not significant in this small patient cohort (Supplementary Fig. 6c, d; Wilcoxon rank-sum). Together, these analyses show an association between the c-Score and DNA repair deficiency. Potentially more clinically relevant, these observations also suggest that additional mechanisms beyond DNA repair deficiency underlie the T cell-inflammation predicted by the c-Score and highlight the potential complementary clinical utility of the c-Score with TMB and markers of DNA repair deficiency.

### Relationship of c-Score, somatic mutations and TMB

**Fig. 6:**
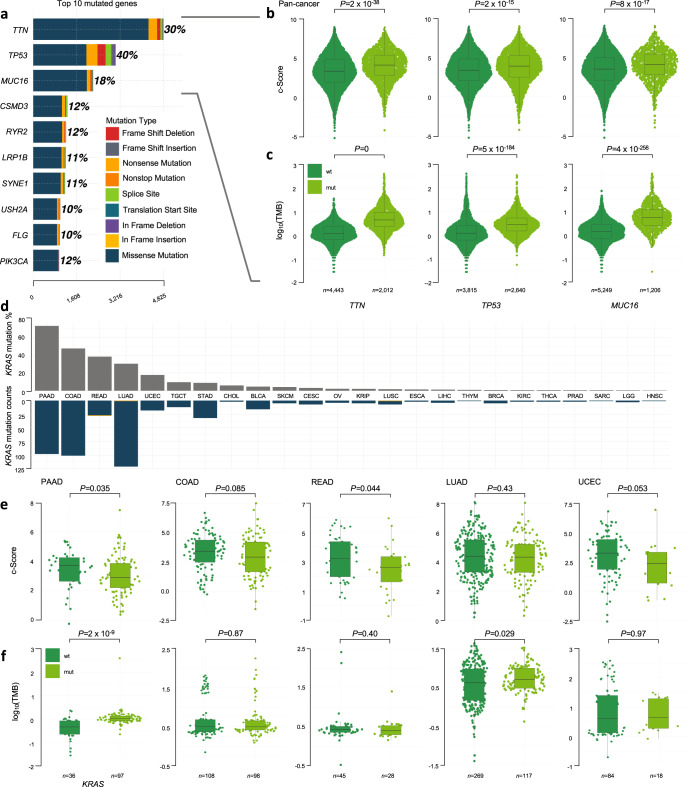
*KRAS* mutation correlations with antitumoral immunity. **a** Histogram of the top 10 somatic gene mutations across the 31 TCGA tumor types. Comparison of (**b**), c-Score and (**c**), TMB in tumors with wild-type (wt) versus mutated (mut) *TTN*, *TP53*, or *MUC16* across the 25 tumor types from TCGA. **d** Overview of somatic *KRAS* mutation status across the cohort (*n* = 6386; *KRAS*-mutated tumors were not identified in MESO), shown as percentage (top) and absolute counts (bottom), with missense representing the majority of mutations. **e** c-Score, and (**f**), TMB in *KRAS*-mutated *versus* wild-type tumors across the top five *KRAS*-mutated tumors across the cohort. Wilcoxon rank-sum test. Median, quartiles, minimum and maximum values are represented by the central line, limits of box, and ends of lines of boxplots shown in (**b**, **c**, **e**, and **f**).

Considering the potential association of oncogenic mutations, beyond DNA repair genes, with cancer immunity, we also investigated the relationship of somatic mutations with T cell-inflammation, as predicted by the c-Score, across TCGA cohort. Among the entire 31 tumor types, the top 10 genes with somatic mutations were *TTN*, *TP53*, *MUC16*, *CSMD3*, *RYR2*, *LRP1B*, *SYNE1*, *USH2A*, *FLG*, and *PIK3CA* (Fig. 6a). We compared the expression of the c-Score in wild-type *versus* mutated tumors for the top three mutated genes. For all three genes, tumors harboring mutations in these genes had increased c-Score expression, including after controlling for tumor type (Fig. 6b). Tumors harboring these mutations also had increased TMB (Fig. 6c).

### Relationship of c-Score, *KRAS* mutations and TMB

Oncogenic *KRAS* mutations commonly occur across cancer types^50^, have been associated with immunosuppressive phenotypes, and may represent a potential mechanism of ICI treatment evasion^24,51,52^. Therefore, we investigated the relationship of *KRAS*-mutated tumors with the c-Score. Pancreatic ductal adenocarcinoma (PAAD) harboured the most *KRAS* mutations (73%), followed by COAD (48%), rectum adenocarcinoma [READ] (38%), LUAD (30%), and UCEC (18%, Fig. 6d). A tumor-agnostic analysis across the 25 TCGA tumor types did not identify a c-Score expression difference between *KRAS* wild-type *versus* mutated tumors (Supplementary Fig. 7a; Wilcoxon rank-sum). Further analysis of the five tumor types with highest prevalence of *KRAS* mutations revealed an association in PAAD and READ, whereby *KRAS*-mutated tumors displayed lower expression of the c-Score *versus KRAS* wild-type tumors (Fig. 6e; MSI status was controlled using MANTIS for READ (*P* = 0.016, multivariate linear regression), while PAAD did not include cases with MSI). A c-Score association with *KRAS*-mutated tumors was not observed in COAD (*P* = 0.085) and LUAD (*P* = 0.43), while there was a trend to a lower c-Score in *KRAS*-mutated UCEC (*P* = 0.053, Wilcoxon rank-sum).

*KRAS*-mutated PAAD and LUAD were found to harbor higher TMB (Fig. 6f; *P* = 2 × 10^−9^, *P* = 0.029, respectively, Wilcoxon rank-sum). However, this association was lost in LUAD when controlling for MANTIS designations. Across tumor types, *KRAS*-mutated tumors had a higher TMB *versus KRAS* wild-type tumors (Supplementary Fig. 7b; *P* = 3 × 10^−22^, Wilcoxon rank-sum; *P* = 0.013 after controlling for MSI status using MANTIS) but was also lost after controlling for tumor type (*P* = 0.58, multivariate linear regression). Although there were more c-Score^hi^ tumors classified as *KRAS* wild-type *versus KRAS*-mutated, this correlation did not reach significance (Supplementary Fig. 7c; *P* = 0.26, Fisher’s exact test). The absence of a significant c-Score^hi^ association with *KRAS* wild-type tumors may be, at least in part, due to a countereffect exerted by a higher mutational load in *KRAS*-mutated tumors, supported by the higher proportion of tumors with TMB ≥ 10 in the *KRAS*-mutated group (Supplementary Fig. 7d; *P* = 1 × 10^−9^, Fisher’s exact test). Taken together, these results support a paradigm whereby genomic drivers, such as mutated *KRAS*, and defects in DNA repair pathways/TMB may have counteracting effects on tumor immunogenicity, where contributions from each process are dictated by additional underlying mechanisms specific to tumor types and their microenvironments.

## Discussion

The c-Score, derived from expression of *CCL4*, *CCL5*, *CXCL9*, and *CXCL10*, identified subpopulations of tumors across cancer types with hallmarks of T cell-inflammation. Tumors with high expression of the c-Score displayed increased transcriptional hallmarks of a T cell-inflamed phenotype, suggesting that c-Score^hi^ tumors have properties of an activated immune response. We found that most histological tumor types had a subpopulation of tumors displaying a high expression of the c-Score, including histological tumor types that have been broadly classified as immune cold. Across cancer types, the proportion of c-Score^hi^ tumors ranged from 0% in pheochromocytoma and kidney chromophobe tumors to 61% in testicular germ cell tumors. T cell-inflammation stratification of tumor types according to c-Score correlated with previously reported antitumor immunity phenotypes and ICI response classifications^27,31,38,48^. Notably, two of the four chemokines (*CXCL9*, *CXCL10*) in the c-Score were present in a 10 gene M1-M2 macrophage signature recently shown to be predictive of ICI therapy outcomes^41^. These observations align with evidence implicating chemokines as putative mediators of tumor-homing in adoptive T cell therapy^53^.

We also show that the c-Score has complementary clinical utility to TMB. In a cohort of 82 patients treated with ICIs^40^, the c-Score^hi^ group exhibited a longer median time to progression and overall survival. We observed a correlation between c-Score^hi^ and TMB^hi^ assignments, with c-Score^hi^TMB^hi^ tumors showing the longest time to progression following ICI treatment initiation. However, the correlation between c-Score^hi^ and TMB^hi^ was imperfect, identifying patients that were c-Score^hi^ while TMB^lo^ with responses to ICI therapy. In addition, the c-Score outperformed TMB for overall survival prediction. While a limitation of this real-world cohort is that patients received different types of ICIs, combined ICIs, or ICIs with chemotherapy, our observations support a role for ICI therapy in patients with tumors characterized by high expression of the c-Score that would otherwise be ineligible for ICI therapy based on TMB stratification alone. The clinical utility of the c-Score as a predictor of ICI response was further validated using the TIDE^25^ and PredictIO^26^ databases.

Since genome-wide tumor mutagenesis may promote T cell-inflammation^20,54^, we evaluated the c-Score relationship with DNA repair mutations. We examined tumors with MSI as a proof of principle since those with MMRD, the pathway resulting in MSI, respond favorably to ICIs^43,54^, and explored whether tumors harboring HRD are also c-Score^hi^. We found tumors with high MSI and HRD scores harbored increased expression of the c-Score, and that a higher proportion of patients with ‘TMB-associated’ tumors (*i.e*., c-Score^hi^ and elevated TMB) had a mutation in a DNA damage repair gene *versus* ‘TMB-unassociated’ tumors. MSI tumors had elevated c-Score expression, and the HRD associations support a role for DNA repair pathway defects in cancer-immunity cycle that extends beyond MMRD. However, the c-Score^hi^ association in HRD tumors was lost when controlling for histological tumor type, suggesting T cell-inflamed phenotypes vary across HRD tumor types.

To examine the relationship of somatic mutations on T cell-inflammation as predicted by the c-Score, we also applied an agnostic approach. We identified the most common mutated genes in TCGA solid tumors and evaluated the c-Score relationship with tumors harboring mutations in these genes. When considering the three most mutated genes (*TTN*, *TP53*, and *MUC16*), we found that tumors with mutations in these genes had elevated c-Score and TMB measurements. Elevated TMB may reflect increased immune activation through a mutational load triggered by genomic instability, rather than an effect of these mutated genes on TMB. Deciphering the mechanisms underlying these observations was beyond the scope of this study and merits further investigation.

The relationship between *KRAS*-mutated tumor genomes and tumor inflammation predicted by the c-Score was also examined. We evaluated this relationship since oncogenic *KRAS* has been implicated as a prognostic and predictive cancer biomarker. When considering cancer immunity, oncogenic *KRAS* may promote immunosuppressive tumor microenvironments^51,52^. Moreover, *KRAS* is mutated in ~25% of all cancers^50^, and in >90% of cases in certain cancers such as pancreatic ductal adenocarcinoma^55^. The immunosuppressive role of oncogenic *KRAS* is multifaceted, directly and indirectly affecting various steps of the cancer-immunity cycle^56–58^. Our observations suggest that, although *KRAS* mutations may contribute to immunosuppressive properties, they do not fully predict tumor inflammation. We propose that tumor T cell-inflammation predicted by increased expression of the c-Score is mediated by mechanisms specific to histological tumor types and their microenvironments, which include the interplay between the immunosuppressive properties of *KRAS* mutations with the immune stimuli resulting from DNA repair deficiencies characterized by elevated TMB and neoantigen tumor loads. In support of this paradigm, *KRAS* mutations did not impart lower c-Scores in lung adenocarcinoma. That is, the immunosuppressive effects of mutated *KRAS* may be offset by a higher TMB in these *KRAS*-mutated tumors. In fact, *KRAS* mutational status has been associated with increased PD-L1 expression in non-small-cell lung cancers (NSCLC)^59^, and anti-PD-1 therapy responses in NSCLC have been correlated with *KRAS* mutations occurring alone or together with *TP53* loss^60^. Contrary to NSCLC, *KRAS* mutations in pancreatic adenocarcinoma are understood to generate an immunosuppressive milieu^61^. To this end, we found increased expression of the c-Score in *KRAS* wild-type *versus* mutated PAAD. Although a higher TMB was observed in *KRAS*-mutated compared to wild-type PAAD, the TMB levels in PAAD rarely meet the ≥10 mutations/megabase threshold predictive of tumor inflammation and ICI response, suggesting the immunosuppressive effects of oncogenic *KRAS* signaling overpower the immune-stimulating features of increased TMB.

In summary, we show that the c-Score identifies tumor subsets with predicted T cell-inflammation and ICI treatment benefits across a wide spectrum of cancers. Our findings suggest that the c-Score is complementary to TMB in predicting ICI therapy clinical outcomes, providing clinical equipoise for its validation in clinical trial settings.

## Methods

### Cohort and description of publicly available TCGA datasets

Datasets were downloaded as described in the DATA AVAILABILITY STATEMENT. Samples with matched RNA (EBPlusPlusAdjustPANCAN_IlluminaHiSeq_RNASeqV2.geneExp.tsv) and MAF data were then filtered (*i.e*., those not passing quality control, or with other annotations advising removal). Only primary tumors were included. Patients with a prior history of cancer or treatments were excluded. Except for CHOL, esophageal carcinoma (ESCA), mesothelioma (MESO), sarcoma (SARC), STAD, TGCT, and thymoma THYM, the prominent histological subtype was selected. Hematological cancers (diffuse large B-cell lymphoma, [DLBC], acute myeloid leukemia [LAML]) were omitted from downstream analyses due to their immunological properties. In cases of multiple aliquots for primary tumors, the first aliquot was selected. In cases of multiple samples per patient, the aliquot with the highest median expression was selected. A final cohort of 6987 patients was assembled for analyses. In Fig. 1, tumor types with clinical response data to ICI^27^ were matched to TCGA nomenclature, with renal cells classified as KIRC and NSCLC classified as lung adenocarcinoma LUAD. The barcodes, c-Scores, and associator designations for all 6987 patients are listed in Supplementary Data File 2.

### Patient enrollment, data collection, processing, and sequencing for the POG570 cohort

This work was approved by and conducted under the University of British Columbia BC Cancer Research Ethics Board (H12-00137, H14-00681), in compliance with the Tri-Council Policy Statement and the FDA regulations (Belmont) and the Good Clinical Practice principles (Helsinki) and approved by the institutional review board. Patients with advanced or metastatic disease gave informed written consent and were enrolled into the POG (NCT02155621) as described previously^40,41,62^. Treatment histories, response, and survival data for the POG cohort were collected retrospectively using the BC Cancer Pharmacy database and chart review. Durable clinical benefit was defined as treatment for greater than 6 months without disease progression^40^. Time to progression was defined as the time from ICI initiation to the date of discontinuation due to progression, and overall survival as the time from ICI initiation to death. Tumor specimens were collected using needle core biopsies, endobronchial ultrasound biopsies, or tissue resection. Solid tumor specimens were snap frozen, while liquid biopsies were spun down into a cell pellet and resuspended. RNA extraction was performed as previously described^41^. Briefly, following pathological review of samples for tumor cellularity, four 50 um sections were pooled in 2.0 mL tubes with 420-600 uL of RLT Plus lysis buffer (Qiagen) and tris (2-carboxyethyl) phosphine, and co-extraction of DNA and RNA was done from 3–11 tubes using an Aline EvoPure kit (Aline Biosciences, R-907-400-C5). Transcriptomes were sequenced to a target of 150–200 million 75-bp end reads on Illumina HiSeq2500 or NextSeq500. Sequencing statistics were performed as previously described^40^. The barcodes and c-Scores for the POG570 and ICI-treated cohorts are listed in Supplementary Data File 3 and Supplementary Data File 4, respectively.

### RNA-seq analyses

Gene identifiers were converted using the *mapIds* function of the AnnotationDbi package (v.1.54.1) and org.Hs.eg.db (v.3.13.0). In cases where multiple matches were found, the first match was used. The c-Score was calculated by taking the mean log_2_(TPM + 0.001) expression of *CCL4*, *CCL5*, *CXCL9*, and *CXCL10* for each patient. c-Score^hi^ and c-Score^lo^ tumors were defined based on samples with ≥3rd (4.835088) or ≤1st (1.940425) quartile expression, respectively. For downstream analyses, tumor types with at least 5 patients per c-Score group were included, resulting in 25 tumor types for subsequent evaluation (*n* = 6455). CIBERSORT scores for leukocyte fractions of TCGA patients were obtained from Thorsson et al.^48^. Duplicate scores for neutrophils and eosinophils were identified, and the first score was used. For single sample Gene Set Enrichment Analysis (ssGSEA), log_2_(TPM + 0.001) data were filtered to remove genes with 0 expressions in greater than 50% of the samples. Genesets used for ssGSEA were chosen based on their representation of processes in the cancer-immunity cycle, including co-stimulation and co-inhibition of antigen presenting cells, major histocompatibility complex I presence, co-stimulation and co-inhibition of T cells, and cytolytic activity^31^, BATF3 dendritic cell presence^7^, and interferon gamma signaling associated with response to ICI^32^. When present, *CCL4*, *CCL5*, *CXCL9*, and *CXCL10* were removed from these genesets prior to analysis by ssGSEA. ssGSEA was calculated using the *gsva* function of the GSVA package (v.1.40.1) as previously described^6^. To display ssGSEA scores on a positive scale in Fig. 2b, arbitrary units (AU) were created as follows: a pseudo-value equal to 1 minus the median value of the ssGSEA score for geneset_*j*_ for the c-Score^lo+med^ group was added to the median value of the ssGSEA score for geneset_*j*_ for the c-Score^lo+med^ and c-Score^hi^ groups, such that the lowest transformed value was 1. This is shown in equations 1 and 2 below:

For geneset_*j*_ and group_*i*_, where *i* = c-Score^hi^ or c-Score^lo+med^,

1 $${{AU}}_{{ji}}={median}{{({ssGSEAscore}}_{j})}_{i}+1-{median}{({{ssGSEAscore}}_{j})}_{y}\,$$

where y = c-Score^lo+med^ specifically, such that the lowest ssGSEA score value is 1:

2 $$\begin{array}{c}{{AU}}_{{jy}}={median}{\left({{ssGSEAscore}}_{j}\right)}_{y}+1-{median}{\left({{ssGSEAscore}}_{j}\right)}_{y}\\ {A.U}_{{jy}}=1\end{array}$$

Statistics were calculated on non-transformed values. For analysis of the POG570 dataset, RNA-seq reads were aligned using STAR73 (v.2.5.2b) and expression was quantified using RSEM74 (v.1.3.0) as TPMs. All final RNA-seq count data are expressed as log_2_(TPM + 0.001). Input indexed files for STAR and RSEM were generated from the hg38 reference genome (http://hgdownload.cse.ucsc.edu/goldenPath/hg38/bigZips/) and gene annotations were based on Ensembl v.85.

### Mutational data processing

For TCGA analysis, MAF files were processed using the maftools package (v.2.8.0). Tumor mutation burden was calculated using the *tmb* function, with a capture size of 35.8 megabases used, as outlined by the *tcgaCompare* function. SNV-derived neoantigen load was obtained for TCGA patients^48^ and was used to corroborate TMB analyses. To compare mutation rates of specific mutation types in DNA repair and fidelity pathways (MMR: *MLH1*, *MSH2*, *MSH6*, *PMS2*; HR: *BRCA1*, *BRCA2*, *PALB2*; DNA replication: *POLE*, *POLD1*), the number of respective mutations, divided by the total number of patients in that group, were used. To compare overall rates of mutations in DNA repair and fidelity pathways, the total number of patients with any mutation in these genes was tallied and compared between groups. Protein-truncating mutations were defined as frameshift deletions, frameshift insertions, nonsense, splice site, translation start site, and nonstop mutations.

### MSI and HRD subtyping and analyses

For TCGA analysis, MSI status was inferred from MANTIS scores as previously calculated^47^, with MANTIS^hi^ patients having a score greater than 0.4. The HRD score was obtained for TCGA patients^48,49^, which represents a combined metric of HRD-Loss of heterozygosity^63^, large-scale state transitions^64^, and a number of telomeric allelic imbalances^65^, and was used to infer an HRD phenotype. HRDScore^lo^ (a proxy for HR proficiency [HRP]) and HRDScore^hi^ (a proxy for HRD) tumor designations were assigned based on third-quartile thresholds of HRDScore values across all 31 tumor types. Given the high prevalence of HRD in BRCA and OV compared to the rest of the cancers, distributions, and quartiles for these tumors were calculated separately. HRDScore^hi^ tumors were defined as those with an HRDScore ≥46 in BRCA and OV, and ≥27 in the rest of the cohort. Following designation as c-Score^lo^, c-Score^med^, and c-Score^hi^, any tumors with high MANTIS or high HRDScores were further classified. Tumors with both high MANTIS and HRDScores were classified as MANTIS^hi^. The final subclassifications were c-Score^lo+med^ (*n* = 3797), c-Score^hi^ (*n* = 1045), HRDScore^hi^ (*n* = 1670), MANTIS^hi^ (*n* = 205), with HRDScore^lo^ and MANTIS^lo^ comprised of both c-Score^lo+med^ and c-Score^hi^ patients. For *KRAS* mutational status analyses, tumors with missing MANTIS/HRDScore values were classified as MANTIS^lo^/HRDScore^lo^. For POG570 dataset, MSI sensor scores were computed as previously described^66^. Scores above 0.2 were considered high. HRDetect scores were computed using a logistic regression model previously described by Davies and colleagues^67^, and implementation can be found at https://github.com/eyzhao/hrdtools^68^.

### Survival and regression analyses

Kaplan–Meier survival analysis was performed for TTP and OS using the R packages *survival* (v.2.42.3) and *survminer* (v.0.4.2). Differences in nonparametric survival functions were assessed across groups using log-rank tests. To ensure sample sizes were large enough for comparison of the chemokine groups, the c-Score^lo^ and c-Score^med^ groups were combined. Cox proportional hazards models were performed using the R packages *survival* (v.2.42.3) and *forest model* (v0.5.0). Log-rank tests were used to calculate *P* values, and in the case of ties, the Efron approximation was used. Linear regression models were used to account for histology in the durable clinical benefit group *versus* no clinical benefit group (NCB). In the case of multivariate analyses using histology, only tumor types with at least three samples were included. Tumor types for the ICI treated cohort are as previously described^40^. For analysis of PanCuRx^20^ and COMPASS datasets^42^, c-Score designations were calculated relative to the sample sets as opposed to cut offs used for TCGA and POG analysis.

### c-Score validation in pan-cancer ICI-treated datasets

The c-Score (*CCL4, CCL5, CXCL9, CXCL10*) was input into the TIDE^25^ and PredictIO^26^ biomarker query (http://tide.dfci.harvard.edu/setquery/, https://predictio.ca/explore/biomarker/request, respectively) using default settings. For PredictIO, both male and female sexes were included, as well as patients that received anti-CTLA4, anti-PD-1, anti-PD-L1, or a combination. Both FPKM and TPM sequencing results were included.

### Statistical analyses

All statistical analyses were done using the R Statistical programming language (v.4.0.4) and select packages from the CRAN and Bioconductor repositories. Specific statistical tests were performed as outlined in the text. Two-tailed *P* values are shown. *P* values were adjusted for multiple tests when necessary, using FDR correction via the Benjamini-Hochberg procedure. Controlling for variables was performed using a multivariate linear regression model.

### Reporting summary

Further information on research design is available in the Nature Research Reporting Summary linked to this article.

### Supplementary information

Supplementary Data Figures 1-7

Supplementary Data Files 1-4

Reporting Summary

## Data Availability

Gene expression for TCGA pan-cancer datasets (TCGA Pan-Cancer (PANCAN)/gene expression RNA-sequencing (RNA-seq)/TOIL RSEM tpm/tcga_RSEM_gene_tpm), containing the log_2_(TPM + 0.001) expression matrix of 10,535 samples across 60,499 features, was downloaded from the University of California Santa Cruz data repository (https://xenabrowser.net/datapages/). Mutation (mc3.v0.2.8.PUBLIC.maf), annotation (merged_sample_quality_annotations.tsv), and clinical data files (TCGA-CDR-SupplementalTableS1.xlsx, clinical_PANCAN_patient_with_followup.tsv) were downloaded from respective manifests and GDC transfer tool (https://gdc.cancer.gov/about-data/publications/pancanatlas, https://gdc.cancer.gov/access-data/gdc-data-transfer-tool). The genomic and transcriptomic sequence datasets, including metadata with library construction and sequencing approaches for the POG570 cohort, have been deposited at the European Genome-phenome Archive (http://www.ebi.ac.uk/ega/) as part of the study EGAS00001001159, and can be downloaded from: http://bcgsc.ca/downloads/POG570/. Specific analysis files for the full cohort and ICI treated cohorts have been previously reported^40,41^.

## Code availability

All bioinformatic analyses were done using the R Statistical programming language (v.4.0.4). The specific packages, parameters, and versions used are outlined in their respective sections found in Methods. The code used in this study, including pipelines for filtering, processing, and analysis, is available upon request.
