# Supplementary material for: Chemokine expression predicts T cell-inflammation and improved survival with checkpoint inhibition across solid cancers
Source: NPJ Precis Oncol. 2023 Aug 9;7:73. doi: 10.1038/s41698-023-00428-2 (PMC10412582; doi:10.1038/s41698-023-00428-2)
Supplement: Supplementary file 1 — Supplementary Data Figures 1-7 [file 41698_2023_428_MOESM1_ESM.pdf]

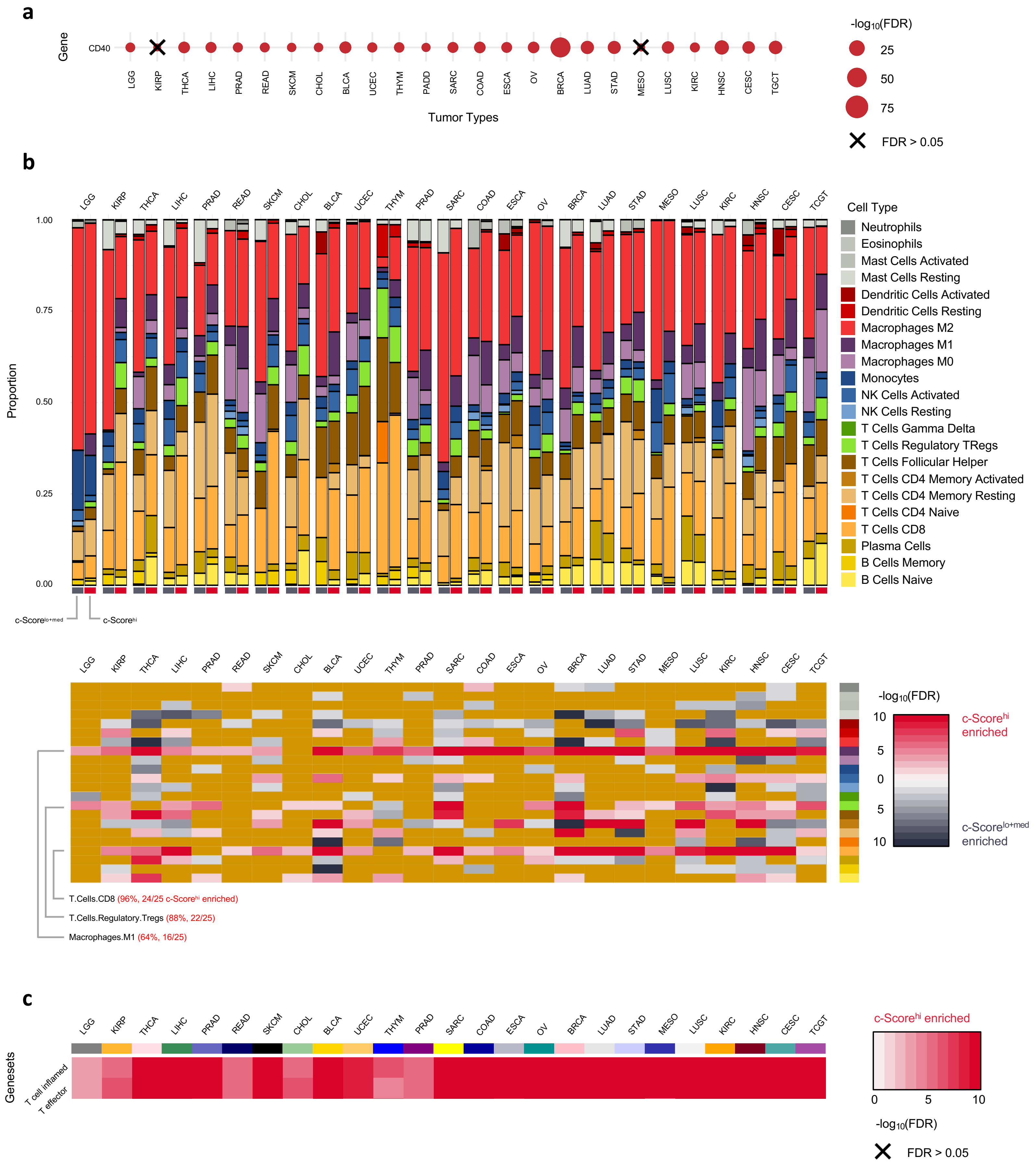

Supplementary Fig. 1 | c-Score^{hi} tumors are enriched in immune-related genes and genesets involved with antitumor immunity. a, Degree of significance in expression of *CD40* between c-Score^{hi} versus c-Score^{lo+med} tumors across 25 tumor types from TCGA. Wilcoxon rank-sum test, FDR adjusted. **b**, Proportion of (top) and degree of significance in (bottom) CIBERSORT scores for 22 immune cell types and **c**, two genesets[34,35] in c-Score^{hi} *versus* c-Score^{lo+med} tumors in this dataset. For **b**, red and blue signify upregulation of expression in c-Score^{hi} *versus* c-Score^{lo+med} groups, respectively. Wilcoxon rank-sum test, FDR adjusted. Gold boxes represent FDR > 0.05.

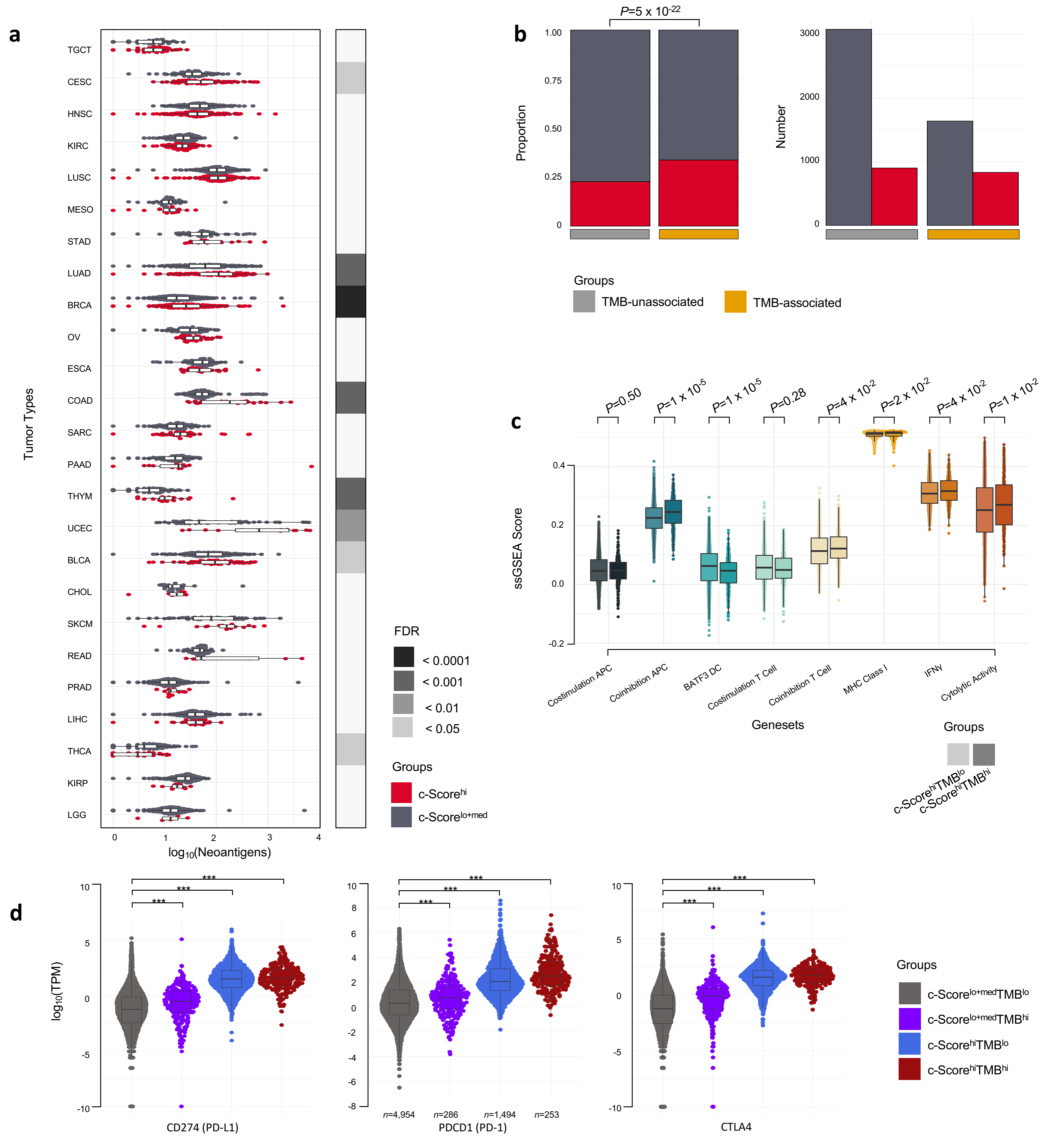

Supplementary Fig. 2 | c-Score status correlation with neoantigen load, TMB and cancer-immunity cycle gene expression. **a**, Association between SNV-derived neoantigen load and expression of the c-Score across 25 cancer types from TCGA. Track displays FDR adjusted *P* values of SNV-derived neoantigen load differences between groups. Wilcoxon rank-sum test, FDR adjusted. **b**, The proportion (left) and absolute number (right) of c-Score^{hi} and c-Score^{lo+med} tumors in 'TMB-unassociated' *versus* 'TMB-associated' in this cohort. Fisher's exact test. **c**, Comparison of ssGSEA scores across eight gene sets involved in the cancer-immunity cycle in c-Score^{hi}TMB^{hi} *versus* c-Score^{hi}TMB^{lo} tumors across all 31 tumor types from TCGA. Boxplot on left (light shade) and right (dark shade) of pairings are TMB^{lo} and TMB^{hi}, respectively. Wilcoxon rank-sum test, FDR adjusted. **d**, Comparison of immune checkpoint genes across all four permutations of TMB and c-Score expression in this cohort. Wilcoxon rank-sum test, FDR adjusted: ***, < 0.001. Median, quartiles, minimum and maximum values are represented by the central line, limits of box, and ends of lines of boxplots shown in **a**, **c**, and **d**.

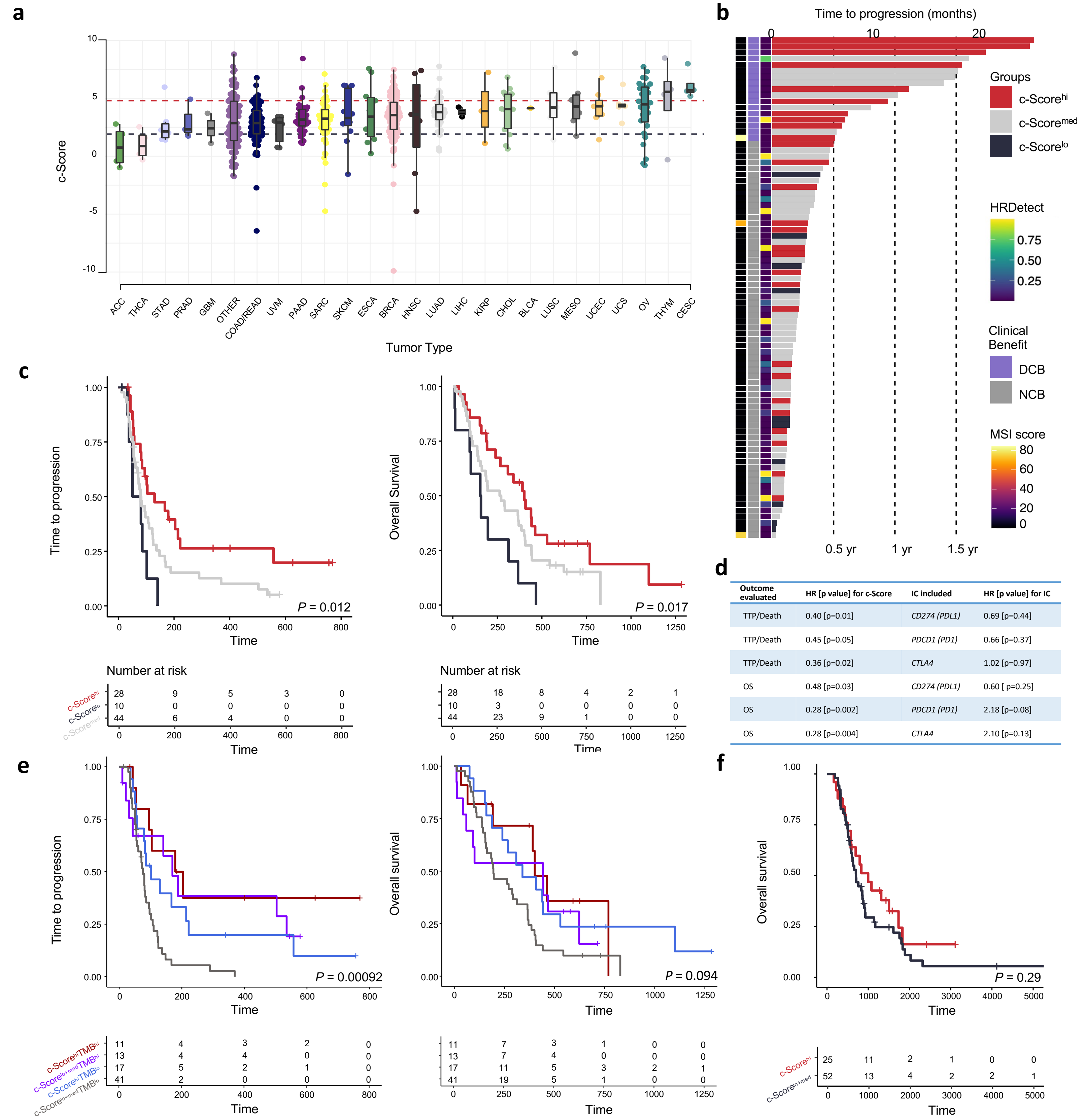

Outcome evaluated	HR [p value] for c-Score	IC included	HR [p value] for IC
TTP/Death	0.40 [p=0.01]	*CD274 (PDL1)*	0.69 [p=0.44]
TTP/Death	0.45 [p=0.05]	*PDCD1 (PD1)*	0.66 [p=0.37]
TTP/Death	0.36 [p=0.02]	*CTLA4*	1.02 [p=0.97]
OS	0.48 [p=0.03]	*CD274 (PDL1)*	0.60 [p=0.25]
OS	0.28 [p=0.002]	*PDCD1 (PD1)*	2.18 [p=0.08]
OS	0.28 [p=0.004]	*CTLA4*	2.10 [p=0.13]

Supplementary Fig. 3 | Distribution and characteristics of c-Score in POG cohort. a, c-Score in POG570 cohort. Tumor types matched to TCGA nomenclature (*n*=559). OTHER refers to a mix of tumor types not specifically matching to TCGA tumor types examined in this manuscript. Dashed lines indicate where thresholds for c-Score^{hi} and c-Score^{lo} groups fall based on TPMs from TCGA data. Median, quartiles, minimum and maximum values are represented by the central line, limits of box, and ends of lines of boxplots shown. **b**, Waterfall plot depicting Time to progression (TTP), split by c-Score groups. HRDetect scores, durable clinical benefit (DCB), and MSIsensor scores are displayed as tracks below. TTP and overall survival (OS) for patients stratified by c-Score group. **d**, Hazard ratios from multivariate cox proportional hazards models for high c-Score, and each IC (high also based on top quartile) from multivariate models run using TMB and tumour type as other variables, for both OS and TTP/death. **e**, TTP and OS for patients stratified by c-Score and TMB group. **f**, Overall survival for patients stratified by c-Score group in the POG dataset starting from date diagnosed with advanced disease (*n*=77). Log-rank test.

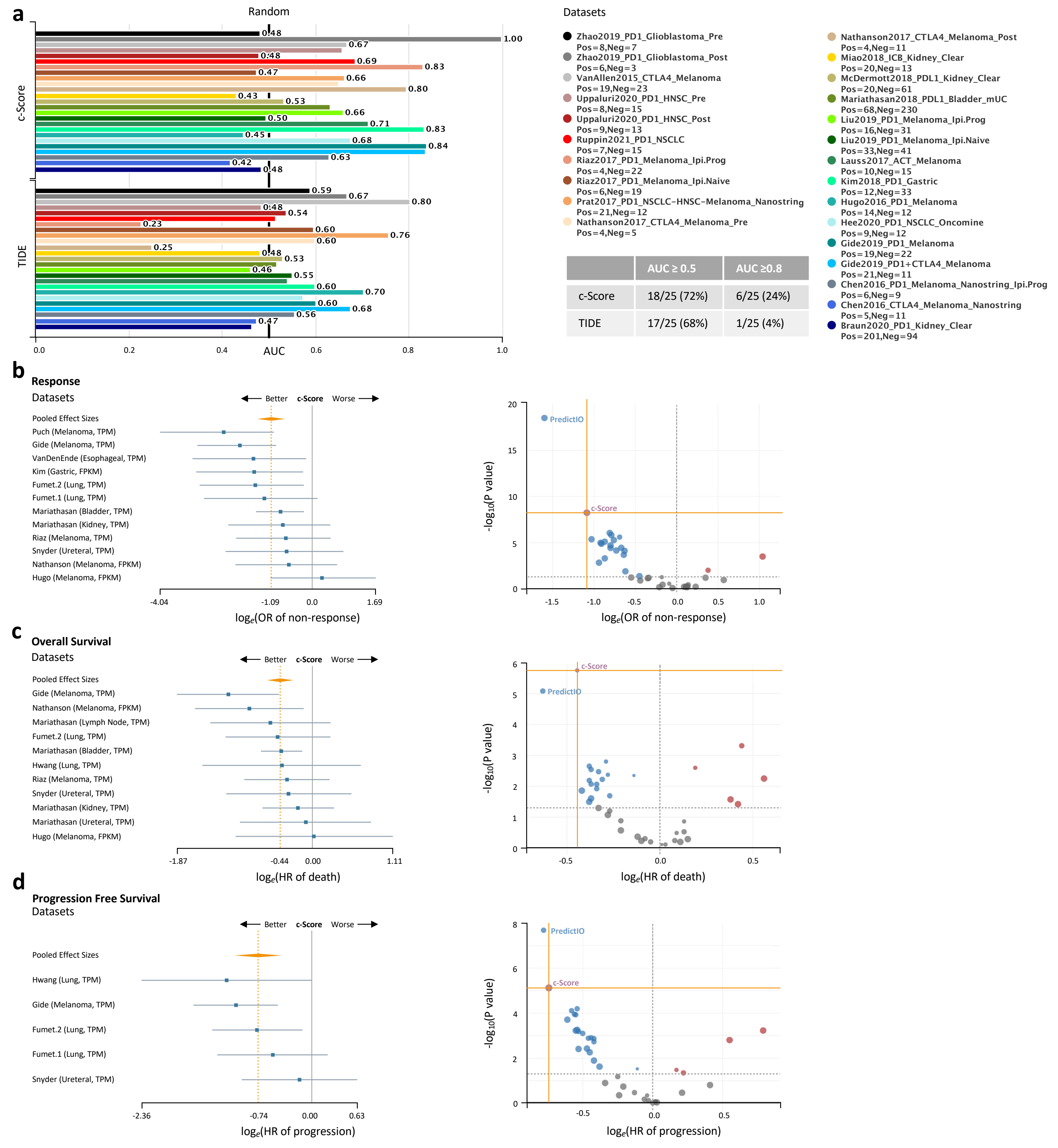

Supplementary Fig. 4 | Validation of c-Score prediction to ICI response across pan-cancer datasets. a, Assessment of the c-Score using the TIDE platform in 25 databases from 19 studies spanning 7 tumor types (*n*=1,290). Area under the curve (AUC) ≥ 0.5 signifies positive value of prediction to ICI response. Table compares c-Score to the TIDE computational framework model. Figures modified from the TIDE platform output[25]. Assessment of the c-Score for **b**, response **c**, overall survival and **d**, progression free survival using the PredictIO platform in 15 databases from 15 studies spanning 8 tumor types (*n*=715). Left panels demonstrate forest plots of each outcome across respective datasets (*n*=529, *n*=559, *n*=188, respectively). Right panels are volcano plots comparing c-Score to 38 published ICI biomarker signatures. PredictIO (blue) and c-Score (purple) are shown. Orange lines delineate -log$_{10}$(P value) and log$_e$(OR) or log$_e$(HR) values obtained from the c-Score. Figures modified from the PredictIO platform output[26]. Error bars represent 95% CI in **b-d**.

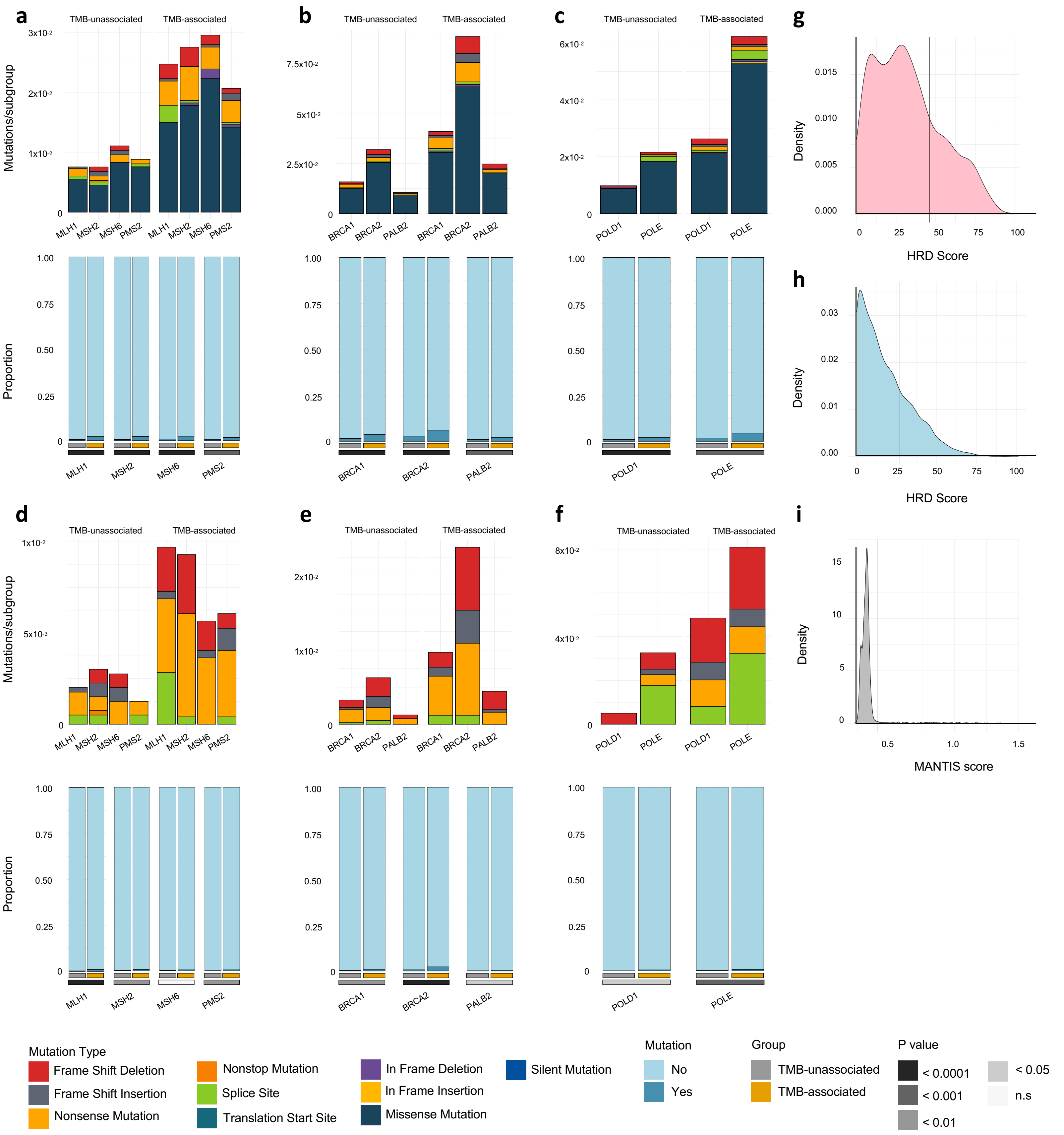

Supplementary Fig. 5 | TMB-associated subgroup has a higher proportion of tumors with mutations in DNA repair pathway mutations. a-c, Comparison of the total number of mutations in 'TMB-unassociated' *versus* 'TMB-associated' groups (top) and proportion of patients with any mutation (bottom) in genes analyzed, namely those involved in **a**, MMRD pathway **b**, HRD pathway, and **c**, *POLD1*/*POLE* genes across 25 tumor types from TCGA. Graphs **d-f** depict protein-truncating mutations. Fisher's exact test. **g**,**h**, Density distribution of HRD scores across **g**, BRCA and OV tumors and **h**, remaining tumor types, excluding MANTIS^{hi} tumors in the cohort. Vertical lines display HRDScore^{hi} cutoffs (46 and 26, respectively). **i**, Density distribution of MANTIS scores across all tumor types, excluding HRDScore^{hi} tumors.

a

MANTIS vs c-Score			
**type**	**rho**	**p.val**	**adj.p.val**
ACC	−0.0616852279	6.23e−01	0.804708333
BLCA	−0.0873396656	2.48e−01	0.549142857
BRCA	0.0319259956	4.56e−01	0.755421053
CESC	−0.0212676478	7.93e−01	0.945500000
CHOL	−0.4030769231	4.22e−02	0.186885714
COAD	0.3516306657	8.03e−07	0.000024893
ESCA	0.0261516577	8.76e−01	0.969857143
GBM	0.0344839551	7.11e−01	0.881640000
HNSC	−0.0005657852	9.93e−01	0.993000000
KICH	−0.0126837537	9.24e−01	0.987724138
KIRC	−0.0017550387	9.77e−01	0.993000000
KIRP	−0.1957108269	2.42e−03	0.025006667
LGG	−0.0585337945	2.10e−01	0.527000000
LIHC	−0.1591339050	1.06e−02	0.082150000
LUAD	0.0387742633	5.64e−01	0.788478261
LUSC	−0.0760576551	4.03e−01	0.755421053
MESO	−0.1808650128	1.95e−01	0.527000000
OV	−0.2086097207	4.14e−02	0.186885714
PAAD	−0.0667688593	5.18e−01	0.777952381
PCPG	−0.1619409315	6.57e−02	0.254587500
PRAD	−0.0571293149	3.08e−01	0.636533333
READ	0.0792626921	5.27e−01	0.777952381
SARC	−0.1401318994	2.21e−01	0.527000000
SKCM	−0.0616206328	5.85e−01	0.788478261
STAD	0.0975704676	1.57e−01	0.486700000
TGCT	−0.3407466890	2.37e−04	0.003673500
THCA	0.0944472558	9.69e−02	0.333766667
THYM	0.0760615063	4.50e−01	0.755421053
UCEC	0.2165994918	4.15e−02	0.186885714
UCS	−0.0391304348	8.56e−01	0.969857143
UVM	0.0860398621	4.63e−01	0.755421053

b

HRDScore vs c-Score		
**rho**	**p.val**	**adj.p.val**
−0.217855171	7.89e−02	1.881462e−01
0.183647349	6.43e−04	5.742750e−03
0.267100727	2.92e−12	4.526000e−11
−0.038609428	5.76e−01	6.865926e−01
0.266759671	1.27e−01	2.187222e−01
0.043309778	5.78e−01	6.865926e−01
0.170637646	5.13e−02	1.325250e−01
−0.120897388	1.85e−01	3.018421e−01
−0.357255997	1.76e−14	5.456000e−13
0.040175686	7.61e−01	8.425357e−01
0.097953045	1.14e−01	2.187222e−01
0.190010847	3.12e−03	1.452571e−02
−0.006770152	8.84e−01	9.134667e−01
−0.038017809	4.99e−01	6.445417e−01
0.135533144	9.05e−03	3.506875e−02
−0.082635135	9.35e−02	2.070357e−01
−0.008570150	9.45e−01	9.450000e−01
0.222405737	2.85e−03	1.452571e−02
−0.189381217	3.91e−02	1.101909e−01
0.084033529	3.42e−01	4.819091e−01
0.156736318	3.28e−03	1.452571e−02
−0.091289868	4.49e−01	6.051739e−01
0.155250186	3.53e−02	1.094300e−01
−0.118904042	2.41e−01	3.557619e−01
−0.094110665	1.13e−01	2.187222e−01
−0.110650164	2.17e−01	3.363500e−01
−0.010524543	8.54e−01	9.128966e−01
0.331932709	7.41e−04	5.742750e−03
−0.274573890	2.94e−02	1.012667e−01
0.212577735	1.23e−01	2.187222e−01
−0.061873615	5.98e−01	6.865926e−01

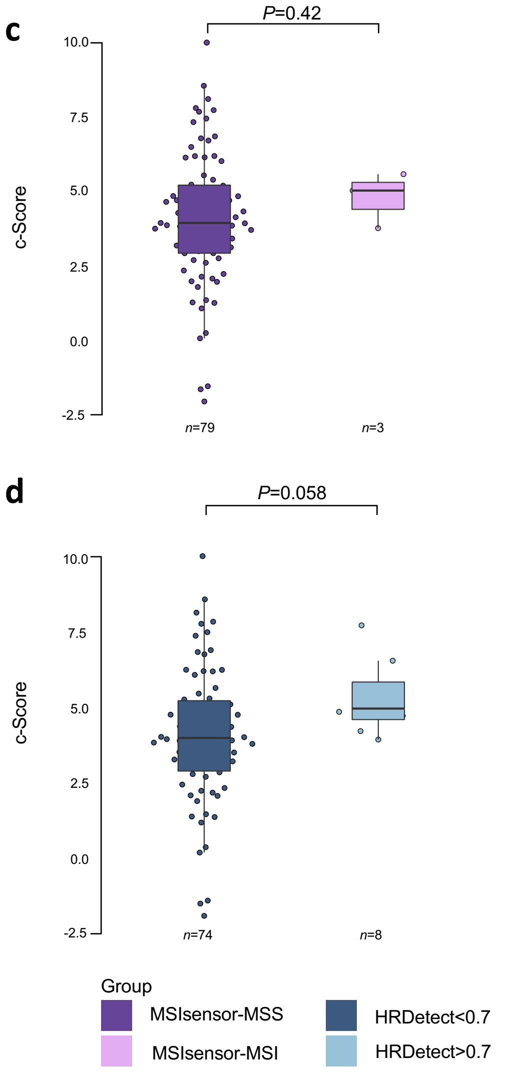

Supplementary Fig. 6 | Associations between c-Score, TMB, and MSI/HRD scores. a, MANTIS Score (*n*=5,047, HRDScore^{hi} tumors omitted), and **b**, HRD score (*n*=6,512, MANTIS^{hi} tumors omitted) across the 31 TCGA tumor types. Spearman correlation, FDR adjusted. Comparison of c-Score in **c**, MSIsensor^{hi} *versus* MSIsensor^{lo} and **d**, HRDetect^{hi} *versus* HRDetect^{lo} tumors. Median, quartiles, minimum and maximum values are represented by the central line, limits of box, and ends of lines of boxplots shown in **c** and **d**.

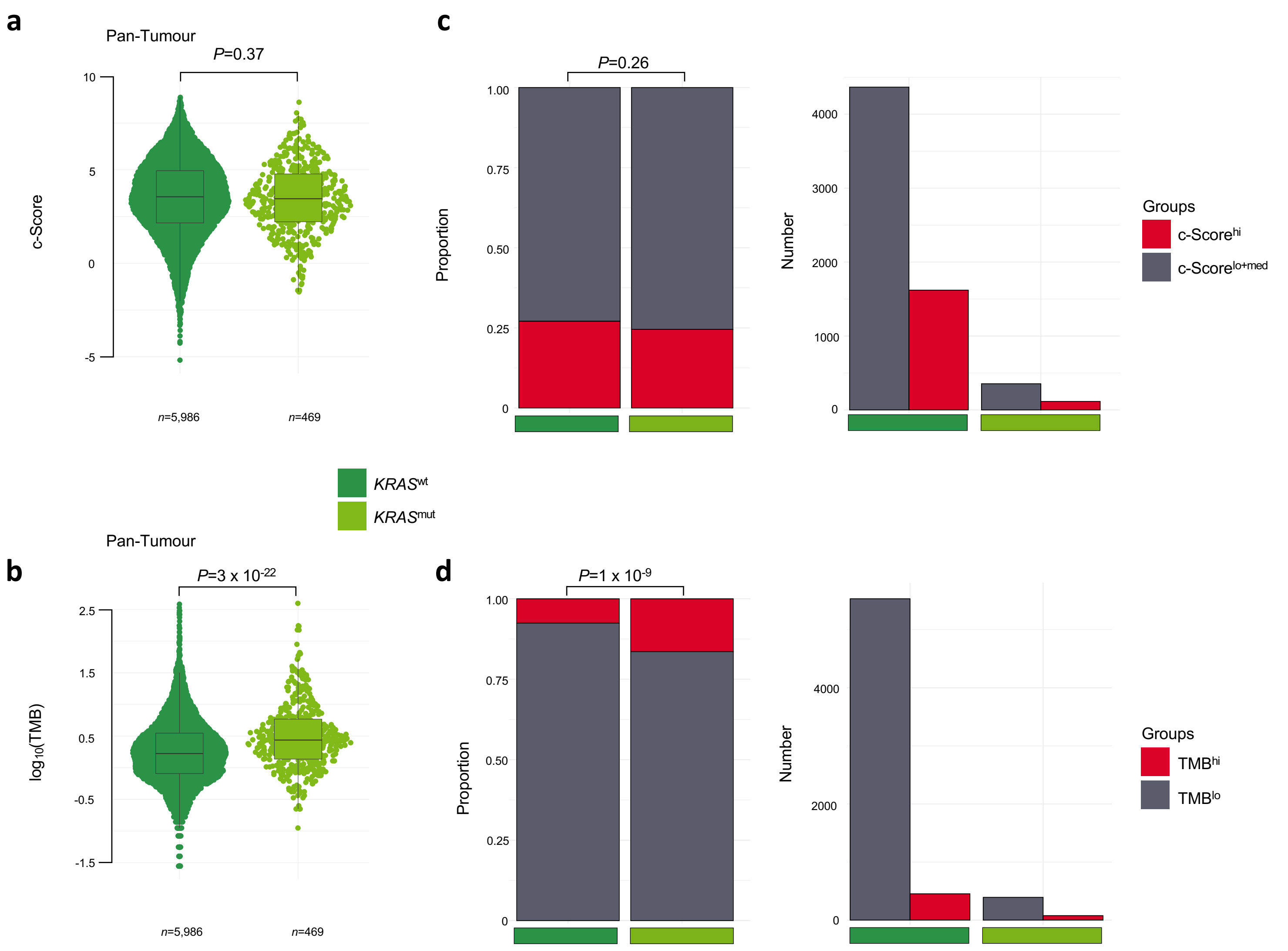

Supplementary Fig. 7 | Comparison of TMB and c-Score in wild-type versus *KRAS*-mutated tumors. a, c-Score and **b**, TMB wild-type *versus* *KRAS*-mutated tumors across the 25 TCGA tumor types. Wilcoxon rank-sum test. **c**, The proportion (left) and absolute number (right) of c-Score^{hi} and c-Score^{lo+med} tumors and **d**, TMB^{hi} and TMB^{lo} tumors in wild-type versus KRAS-mutated tumors in this cohort. Fisher's exact test. Median, quartiles, minimum and maximum values are represented by the central line, limits of box, and ends of lines of boxplots shown in **a** and **b**.
