# Supplementary material for: Chemokine expression predicts T cell-inflammation and improved survival with checkpoint inhibition across solid cancers
Source: NPJ Precis Oncol. 2023 Aug 9;7:73. doi: 10.1038/s41698-023-00428-2 (PMC10412582; doi:10.1038/s41698-023-00428-2)
Supplement: Supplementary file 3 — Reporting Summary [file 41698_2023_428_MOESM3_ESM.pdf]

nature portfolio

Corresponding author(s): George Zogopoulos

Last updated by author(s): Apr 24, 2023

Reporting Summary

Nature Portfolio wishes to improve the reproducibility of the work that we publish. This form provides structure for consistency and transparency in reporting. For further information on Nature Portfolio policies, see our Editorial Policies and the Editorial Policy Checklist.

Statistics

For all statistical analyses, confirm that the following items are present in the figure legend, table legend, main text, or Methods section.

n/a	Confirmed	
☐	☒	The exact sample size (n) for each experimental group/condition, given as a discrete number and unit of measurement
☐	☒	A statement on whether measurements were taken from distinct samples or whether the same sample was measured repeatedly
☐	☒	The statistical test(s) used AND whether they are one- or two-sided<br>*Only common tests should be described solely by name; describe more complex techniques in the Methods section.*
☐	☒	A description of all covariates tested
☐	☒	A description of any assumptions or corrections, such as tests of normality and adjustment for multiple comparisons
☐	☒	A full description of the statistical parameters including central tendency (e.g. means) or other basic estimates (e.g. regression coefficient) AND variation (e.g. standard deviation) or associated estimates of uncertainty (e.g. confidence intervals)
☐	☒	For null hypothesis testing, the test statistic (e.g. F, t, r) with confidence intervals, effect sizes, degrees of freedom and P value noted<br>*Give P values as exact values whenever suitable.*
☒	☐	For Bayesian analysis, information on the choice of priors and Markov chain Monte Carlo settings
☒	☐	For hierarchical and complex designs, identification of the appropriate level for tests and full reporting of outcomes
☐	☒	Estimates of effect sizes (e.g. Cohen's d, Pearson's r), indicating how they were calculated

Our web collection on statistics for biologists contains articles on many of the points above.

Software and code

Policy information about availability of computer code

Data collection	Mutation (mc3.v0.2.8.PUBLIC.maf), annotation (merged_sample_quality_annotations.tsv), and clinical data files (TCGA-CDR-SupplementalTableS1.xlsx, clinical_PANCAN_patient_with_followup.tsv) for TCGA datasets were downloaded from respective manifests using the GDC transfer tool (https://gdc.cancer.gov/about-data/publications/pancanatlas, https://gdc.cancer.gov/access-data/gdc-data-transfer-tool).<br><br>CIBERSORT, MANTIS scores, HRD scores, and SNV-derived neoantigen load for TCGA datasets was obtained from respective studies, as detailed in Methods.<br><br>All other data collection and downstream processing for datasets was done using the R Statistical Programming language (v.4.0.4), as described in Methods.
Data analysis	All data analysis was done using the R Statistical Programming language (v.4.0.4) and respective packages from the CRAN and Bioconductor repositories, as outlined below.<br><br>For TCGA dataset:<br>Conversion of gene identifiers:<br>mapIds function of AnnotationDbi (v.1.54.1), org.Hs.eg.db (v.3.13.0)<br><br>Single sample Gene Set Enrichment Analysis:<br>gsva function of GSVA (v.1.40.1)

Tumor mutation burden calculation:
tmb function of maftools (v.2.8.0)

For POG dataset:
Survival analysis:
survival (v.2.42.3), survminer (v.0.4.2)

Evaluation of c-Score using TIDE and PredictIO databases were performed on their respective platforms as described in Methods:
TIDE: http://tide.dfci.harvard.edu/setquery/
PredictIO: https://predictio.ca/explore/biomarker/request

For manuscripts utilizing custom algorithms or software that are central to the research but not yet described in published literature, software must be made available to editors and reviewers. We strongly encourage code deposition in a community repository (e.g. GitHub). See the Nature Portfolio guidelines for submitting code & software for further information.

Data

Policy information about availability of data

All manuscripts must include a data availability statement. This statement should provide the following information, where applicable:

- Accession codes, unique identifiers, or web links for publicly available datasets
- A description of any restrictions on data availability
- For clinical datasets or third party data, please ensure that the statement adheres to our policy

TCGA datasets analysed in the present study have been obtained as described above (DNA: https://gdc.cancer.gov/about-data/publications/pancanatlas, RNA: https://xenabrowser.net/ datapages/).

The genomic and transcriptomic sequence datasets, including metadata with library construction and sequencing approaches for the POG570 cohort, have been deposited at the European Genome-phenome Archive (http://www.ebi.ac.uk/ega/) as part of the study EGAS00001001159, and can be downloaded from: http://bcgsc.ca/downloads/POG570/. Specific analysis files for the full cohort and ICI treated cohorts have been previously reported42,43.

Research involving human participants, their data, or biological material

Policy information about studies with human participants or human data. See also policy information about sex, gender (identity/presentation), and sexual orientation and race, ethnicity and racism.

Reporting on sex and gender	Gender information for participants in TCGA (n=6,987) is not available. For this pan-cancer analysis, the associations of the c-Score on immune-related measures including gene and geneset expression, and their relationships with potential mechanisms of neoantigenicity, including HRD status, MSI status, and TMB, was assessed. As such, further subgrouping of patients on demographic variables, including sex, was outside the scope of this study. Given the limited sample size of ICI-treated POG patients (n=82), further sugrouping was not performed for this and the latter reason.
Reporting on race, ethnicity, or other socially relevant groupings	The objectives of this study were to asses the association of the c-Score with immune-related measures and potential mechanisms of neoantigenicity. Accordingly, assessment of the role of race, ethnicity, or other socioeconomic parameters was outside the scope of this study.
Population characteristics	For TCGA, the study population included 31 tumor types from 6,987 treatment naive patients without a prior history of previous cancer and no metastatic disease, of the following histological types: ACC (n=72), 352 BLCA, (n=352), BRCA (n=720), CESC (n=227), CHOL (n=34), COAD (n=206), ESC (n=151), GBM (n=125), HNSC (n=447), KICH (n=60), KIRC (n=306), KIRP (n=242), LGG (n=479), LIHC (n=332), LUAD (n=386), LUSC (n=420), MESO (n=69), OV (n=187), PAAD (n=133), PCPG (n=143), PRAD (n=365), READ (n=73), SARC (n=191), SKCM (n=99), STAD (n=363), TGCT (n=126), THCA (n=329), THYM (n=116), UCEC (n=102), UCS (n=57), and UVM (n=75). For the ICI-treated POG datasets, 19 tumor types from 82 patients previously treated with ICI therapy were analyzed, with histological types including: ACC (n=2), AECA (n=2), BRCA (n=12), CHOL (n=3), COLO (n=6), ESCA (n=1), GCT (n=1), (n=HNSC (n=4), KDNY (n=2), LUNG (n=25), LYMP (n=1), OV (n=1), PANC (n=2), SARC (n=5), SKCM (n=7), STAD (n=2), THYM (n=1), UCEC (n=3), UVM (n=2). Remaining datasets and abbreviations are described in text.
Recruitment	Patients with advanced or metastatic disease gave informed written consent and were enrolled into the POG study (NCT02155621). Further details can be found in Methods.
Ethics oversight	University of British Columbia BC Cancer Research Ethics Board (H12-00137, H14-00681)

Note that full information on the approval of the study protocol must also be provided in the manuscript.

Field-specific reporting

Please select the one below that is the best fit for your research. If you are not sure, read the appropriate sections before making your selection.

☒ Life sciences ☐ Behavioural & social sciences ☐ Ecological, evolutionary & environmental sciences

For a reference copy of the document with all sections, see nature.com/documents/nr-reporting-summary-flat.pdf

Life sciences study design

All studies must disclose on these points even when the disclosure is negative.

Sample size	Sample sizes were obtained following filtering of datasets as described below. The final sample sizes after filtering and exclusion, when applicable, was n=6,987 (TCGA), n=559 (POG570), n=82 (ICI-treated POG), 1,650 (TIDE+PredictIO).
Data exclusions	For the TCGA analysis, patients with metastatic disease, prior history of cancer, or prior history of treatment, and samples without matching RNA or DNA data and not passing quality control, were excluded from downstream analyses. For ICI-treated POG, patients previously treated with ICI were included for downstream analyses.
Replication	Prediction of T cell-inflammation using the c-Score seen in an independent cohort of pancreatic tumors was validated across 6,987 tumors from TCGA. Association of ICI response with the c-Score seen in the ICI-treated POG datasets was validated using two databases spanning 28 studies from 1,650 samples.
Randomization	Given the nature of classification, samples were not randomized. Rather samples were grouped into c-Score or TMB classifications based on mean expression of CCL4, CCL5, CXCL9, and CXCL10 or mutational load, as described in Methods.
Blinding	Given the nature of analyses, this retrospective association study did not require blinding.

Reporting for specific materials, systems and methods

We require information from authors about some types of materials, experimental systems and methods used in many studies. Here, indicate whether each material, system or method listed is relevant to your study. If you are not sure if a list item applies to your research, read the appropriate section before selecting a response.

Materials & experimental systems

n/a	Involved in the study	
☒	☐	Antibodies
☒	☐	Eukaryotic cell lines
☒	☐	Palaeontology and archaeology
☒	☐	Animals and other organisms
☐	☒	Clinical data
☒	☐	Dual use research of concern
☒	☐	Plants

Methods

n/a	Involved in the study	
☒	☐	ChIP-seq
☒	☐	Flow cytometry
☒	☐	MRI-based neuroimaging

Clinical data

Policy information about clinical studies

All manuscripts should comply with the ICMJE guidelines for publication of clinical research and a completed CONSORT checklist must be included with all submissions.

Clinical trial registration	NCT02155621
Study protocol	https://clinicaltrials.gov/ct2/show/NCT02155621
Data collection	Treatment histories, response, and survival data for the POG cohort were collected retrospectively using the BC Cancer Pharmacy database and chart review. Tumor specimens were collected using needle core biopsies, endobronchial ultrasound biopsies, or tissue resection. Solid tumor specimens were snap frozen, while liquid biopsies were spun down into a cell pellet and resuspended.<br><br>All other data used is publicly available and can be accessed from respective sources, as outlined in Methods.
Outcomes	This manuscript reports the analysis of a 4-chemokine signature as a predicitve biomarker of T cell-inflammation and immune checkpoint inhibition response using datasets with transcriptomic and matching clinical outcome data. As such, primary and secondary outcomes of the POG clinical trial are not reported here.
